# Assessing the role of membrane lipids in the action of ruthenium(III) anticancer compounds

**DOI:** 10.3389/fmolb.2022.1059116

**Published:** 2023-01-04

**Authors:** Radoslaw Starosta, Telma C. Santos, Andreia F. Dinis de Sousa, Maria Soledade Santos, M. Luisa Corvo, Ana Isabel Tomaz, Rodrigo F. M. de Almeida

**Affiliations:** ^1^ Faculty of Chemistry, University of Wroclaw, Wroclaw, Poland; ^2^ Centro de Química Estrutural, Institute of Molecular Sciences, Departamento de Química e Bioquímica, Faculdade de Ciências, Universidade de Lisboa, Lisbon, Portugal; ^3^ Research Institute for Medicines (iMed.ULisboa), Faculdade de Farmácia, Universidade de Lisboa, Lisbon, Portugal

**Keywords:** biomimetic lipid membrane, ruthenium complexes, membrane leakage, lipid domains, fluorescence spectroscopy, membrane dipole potential

## Abstract

This work addresses the possible role of the cell membrane in the molecular mechanism of action of two *salan*-type ruthenium complexes that were previously shown to be active against human tumor cells, namely [Ru(III)(**L1**)(PPh_3_)Cl] and [Ru(III)(**L2**)(PPh_3_)Cl] (where **L1** is 6,6′-(1*R*,2*R*)-cyclohexane-1,2-diylbis(azanediyl)bis(methylene)bis(3-methoxyphenol); and **L2** is 2,2′-(1*R*,2*R*)-cyclohexane-1,2-diylbis(azanediyl)bis(methylene)bis(4-methoxyphenol)). One-component membrane models were first used, a disordered fluid bilayer of dioleoylphosphatodylcholine (DOPC), and an ordered rigid gel bilayer of dipalmitoylphosphatidylcholine. In addition, two quaternary mixtures of phosphatidylcholine, phosphatidylethanolamine, sphingomyelin and cholesterol were used to mimic the lipid composition either of mammalian plasma membrane (1:1:1:1 mol ratio) or of a cancer cell line membrane (36.2:23.6:6.8:33.4 mol ratio). The results show that both *salan* ligands **L1** and **L2** bind relatively strongly to DOPC bilayers, but without significantly affecting their structure. The ruthenium complexes have moderate affinity for DOPC. However, their impact on the membranes was notable, leading to a significant increase in the permeability of the lipid vesicles. None of the compounds compromised liposome integrity, as revealed by dynamic light scattering. Fluorescence spectroscopy studies revealed changes in the biophysical properties of all membrane models analyzed in the presence of the two complexes, which promoted an increased fluidity and water penetration into the lipid bilayer in the one-component systems. In the quaternary mixtures, one of the complexes had an analogous effect (increasing water penetration), whereas the other complex reorganized the liquid ordered and liquid disordered domains. Thus, small structural differences in the metal ligands may lead to different outcomes. To better understand the effect of these complexes in cancer cells, the membrane dipole potential was also measured. For both Ru complexes, an increase in the dipole potential was observed for the cancer cell membrane model, while no alteration was detected on the non-cancer plasma membrane model. Our results show that the action of the Ru(III) complexes tested involves changes in the biophysical properties of the plasma membrane, and that it also depends on membrane lipid composition, which is frequently altered in cancer cells when compared to their normal counterparts.

## 1 Introduction

Drug-membrane interactions are increasingly recognized as one of the most important pharmacological features, playing an important role in drugs biological activity (Lucio et al., 2010; Andrade et al., 2021). For drugs with intracellular targets, the plasma membrane can be viewed as a barrier that needs to be efficiently crossed (Bunea et al., 2020; Sharifian Gh, 2021), preferably with some degree of selectivity for the target cells, for example, cancer cells over healthy ones. The plasma membrane, its lipids, and their biophysical properties, however, are increasingly viewed as an important drug target as well. The drug can act mainly by affecting membrane organization or even compromising its integrity, or it can have a specific intracellular target, but the drug activity cannot be explained solely by its effect on a specific molecular target–the effects on the plasma membrane may also contribute to the drug biological activity and be part of complex mechanisms of action (Lucio et al., 2010; Ingólfsson et al., 2014). Biological membranes are highly complex with a dynamic composition that can comprise hundreds of different lipid and proteins. Moreover, these components are not randomly distributed in the plane of the membrane, but rather they are spatio-temporally organized in membrane domains differing in composition, properties and carrying out specific functions (Marquês et al., 2015). Membrane lipid domains, such as the so-called lipid rafts, with their unique composition and biophysical properties, have crucial roles in cell signaling and sorting (Sezgin et al., 2017). Changes in those properties may have vast implications in defining cellular fate, and therefore are intimately related to cancer conditions (Vona et al., 2021). In fact, it is known that lipid composition and organization is markedly different in cancer cells *versus* non-cancer counterparts (Barceló-Coblijn et al., 2011; Bestard-Escalas et al., 2020; Maja et al., 2022). Thus, membrane lipids are emerging as key targets of novel anticancer therapeutics which can be designed to change membrane biophysical properties, either directly or through alterations in lipid metabolism (Barceló-Coblijn et al., 2011; Czyz et al., 2013; Herrera et al., 2017).

The imprinting of particular biophysical properties on the membrane through the combination of multiple lipid species in specific proportions is of major importance for membrane compartmentalization. Indeed, lipids tend to cluster together or to segregate into different phases, therefore creating domains with different size, diffusion properties, fluidity, thickness, surface charge and membrane dipole potential, thus influencing protein function and membrane interaction (Marquês et al., 2015). Small amphiphiles with moderate to strong ability to partition to the membrane, can cause mild to drastic effects on membrane organization (Andrade et al., 2021). The presence of such compounds can change the surface charge, curvature or elasticity of the membrane, its dipole potential, affect the H-bonding network at the membrane surface, disrupt interactions between different types of lipids and lead to membrane fluidization or, by providing additional sites for interaction, hinder the mobility of the lipids and rigidify certain areas of the membrane (Herrera et al., 2017; Fanani et al., 2022). As a consequence, externally added compounds may alter the permeability of the lipid bilayer (Carreira et al., 2017; Pereira-Leite et al., 2020), and/or affect the size, fraction and composition of the different types of domains coexisting in the membrane (Czyz et al., 2013; Filipe et al., 2018; Pereira-Leite et al., 2020).

In cancer chemotherapy, platinum-based agents are the only metallodrugs approved for therapeutic application worldwide. Despite their well-known systemic toxicity and resistance issues, they still stand out for how often they are prescribed, incorporating about 50% of all oncologic treatments (alone or in combination therapy) (Kenny et al., 2017). Research on metallodrugs with the aim of fulfilling the requirements of high activity with a more tolerable pharmacological profile and better selectivity have extended metal-based chemotherapeutics to non-platinum compounds (Leon et al., 2016; Valente et al., 2021). Among these, ruthenium-based compounds have steadily shown great potential with lower systemic toxicity, a wider spectrum of response, inherent selectivity for cancer cells (in some cases) and different modes of action compared to Pt drugs, partly because ruthenium compounds seem to exert their effect through multiple targets (Kenny and Marmion, 2019; Lee et al., 2020; PragtiKundu and Mukhopadhyay, 2021; Valente et al., 2021; Katheria, 2022).

To date, three Ru(III) complexes have progressed into Phase I/II clinical trials, namely NAMI-A (Alessio and Messori, 2018) (now suspended), KP1019 (replaced later by its more soluble sodium salt NKP1339 (Alessio and Messori, 2019), and more recently a Ru(II) complex TLD1433 (in Phase Ib) that was specifically designed for photodynamic therapy (PDT) (Monro et al., 2019). NAMI-A and KP1019 attracted much interest due to their different biological effects despite their similar structure. Both have weak-binding monodentate chloride ligands making them too prone to hydrolysis, which has hindered their progress into an effective drug. (Riccardi et al., 2017; Alessio and Messori, 2019). Currently, among the multitude of different families of ruthenium compounds developed as anti-cancer prospective drugs, the great majority is based on Ru(II) compounds, with most research on Ru(III) being restrained to NAMI-like or KP1910-like complexes with several labile ligands in the coordination environment.

The successful use of a tetradentate chelating ligand such as *salen*/*salan*-like structures to bind Ru in the development of ruthenium catalysts, gathered interest as to their possible therapeutic properties. *Salen* is an acronym for *N*,*N*′-bis(salicylidene)-1,2-ethylenediamine, the prototype of the class; *salan* refers to its tetrahydro-analogue (see Abbreviations List at the start of this paper), the reduced derivative of the former, more flexible and also offering an N_2_O_2_ binding mode). The first report on Ru(III) complexes exhibiting a *salan*-like structure with methoxy-substituted salicylaldehydes and 1,2-diaminocyclohexane ((*R*,*R*)-isomer) as the diamine moiety disclosed the excellent activity of these Ru(III) complexes in human cancer cells, and their interest as a new Ru(III) family of prospective metallodrugs (Domotor et al., 2017). Despite their structural resemblance, the first studies on their mode of action indicated that cell cycle and cell morphology were affected differently upon exposure to each compound, which suggested the possible involvement of several (different) targets in their action (Matos et al., 2013).

**FIGURE 1:**
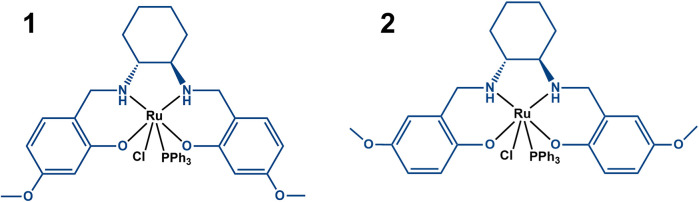
Scheme of complexes **1** ([Ru^III^(**L1)**(PPh_3_)Cl]) and **2** {[Ru^III^(**L2)**(PPh_3_)Cl]}, where **L1** ≡ [6,6′-(1*R*,2*R*)-cyclohexane-1,2-diylbis(azanediyl)bis(methylene)bis(3-methoxyphenol)) and **L2** ≡ (2,2′-(1*R*,2*R*)-cyclohexane-1,2-diylbis(azanediyl)bis(methylene)bis(4-methoxyphenol)] (both in blue) and PPh_3_ is triphenylphosphane.

Surprisingly, despite intense research, mechanisms underlying the action of ruthenium complexes are still not completely understood. Several anti-cancer active ruthenium compounds are now known to preferably accumulate in the membrane rather than undergoing extensive cell uptake, although this is highly dependent on the ligand set, and preliminary studies suggested that it could be the case of these Ru(III)-*salan* complexes as well (Valente et al., 2021). Thus, in this work we studied the possible role of the membrane on the molecular mechanism of action of these two Ru(III)-*salan* complexes (Figure 1), which we have previously shown to be active against human tumor cells (Matos et al., 2013; Domotor et al., 2017).

For the studies presented herein, several membrane model systems were chosen, ranging from one-component to four-component models. The two simple one-component systems are in lipid bilayer phases with very different fluidity at room temperature (Davis, 1979; Stubbs et al., 1981; Gratton and Parasassi, 1995), a disordered fluid phase (1,2-dioleoyl-*sn*-glycero-3-phosphocholine, DOPC), and an ordered gel phase (1,2-dipalmitoyl-*sn*-glycero-3-phosphocholine, DPPC). An anionic two-component mixture of 1,2-dimyristoyl-*sn*-glycero-3- phosphocholine (DMPC) with 1,2-dimyristoyl-*sn*-glycero-3-phosphoglycerol (DMPG) at a 7:3 mol ratio was also used (Gonçalves et al., 2017). The more complex models, containing four representative lipids of the mammalian plasma membrane, namely, 1-palmitoyl-2-oleoyl-*sn*-glycero-3-phosphocholine (POPC), 1- palmitoyl-2-oleoyl-*sn*-glycero-3-phosphoethanolamine (POPE), sphingomyelin (SM) and cholesterol (Chol), were used to mimic either the “canonical” composition of mammalian plasma membranes (POPC:POPE:SM:Chol 1:1:1:1 mol ratio) or the composition of U-118 glioma cell membrane (POPC:POPE:SM:Chol 36.2:23.6:6.8:33.4 mol ratio) (Barceló-Coblijn et al., 2011; Khmelinskaia et al., 2014).

## 2 Materials and methods

### 2.1 Reagents and materials

The compounds used in this study, both ligands and complexes, were obtained in a previous work (Matos et al., 2013; Domotor et al., 2017). Solvents of spectroscopic grade were purchased from VWR International or Fluka. Throughout this work we used three buffer systems:

*buffer 1*: Hepes: 10 mM, pH 7.4, DMSO: 2% (V:V), NaCl: 150 mM
*buffer 2*: Hepes: 10 mM, pH 7.4, DMSO: 2% (V:V)
*buffer 3*: Hepes: 10 mM, pH 7.4, DMSO: 5% (V:V).

While ligands were stable in all buffers (see Supplementary Figure S1, S2), complexes **1** and **2** precipitate slowly in *buffer 1* (Supplementary Figure S3 shows the spectroscopic data for complex **1**). Therefore, all the experiments for the Ru(III) complexes were performed in *buffer 2* and *buffer 3*.

The lipids DOPC, DPPC, POPC, DMPG, DMPC and chicken egg SM were purchased from Lipoid (NJ, United States). POPE and Chol were purchased from Merck.

The membrane probes: 1,6-diphenyl-1,3,5-hexatriene (DPH) and 4-(2-(6-(dibutylamino)-2-naphthalenyl)ethenyl)-1-(3-sulfopropyl)pyridinium hydroxide inner salt (di-4-ANEPPS), were acquired from Thermo Fisher Scientific. 5(6)-carboxyfluorescein (CF) was acquired from Merck. The concentrations of membrane probes in methanol solution were determined spectrophotometrically using their molar absorption coefficients: ε(DPH, 350 nm, MeOH) = 88,000 M^−1^cm^−1^, ε(di-4-ANEPPS, 497 nm, MeOH) = 42,000 M^−1^cm^−1^ (Haugland, 1996). PPh_3_ and [Ru(PPh_3_)_3_Cl_2_] were purchased from Sigma-Aldrich (St Louis, MO, United States). **L1** (6,6′-(1*R*,2*R*)-cyclohexane-1,2-diylbis(azanediyl)bis(methylene)bis(3-methoxyphenol)) and **L2** (2,2′-(1*R*,2*R*)-cyclohexane-1,2-diylbis(azanediyl)bis(methylene)bis(4-methoxyphenol)) were synthesized from 1,2-cyclohexanediamine and 2-hydroxy-4-methoxybenzaldehyde or 2-hydroxy-5-methoxybenzaldehyde, respectively (Merck) (Matos et al., 2013). The complexes **1** {[Ru^III^(**L1**)(PPh_3_)Cl]} and **2** {[Ru^III^(**L2**)(PPh_3_)Cl]} were synthesized as described previously (Matos et al., 2013) in the reactions of [Ru(PPh_3_)_3_Cl_2_] with **L1** and **L2**, respectively.

### 2.2 Liposome preparation

Stock solutions of lipids were prepared in chloroform and their concentrations were determined by the Rouser method (Rouser et al., 1970) each time prior to liposome preparation, except for cholesterol which weight was measured directly in an ultra-analytical balance. The solvent was removed by evaporation under a mild stream of nitrogen followed by overnight drying under vacuum. The lipid films obtained were hydrated with the appropriate buffer to the desired final concentrations. Next, seven vortex/freeze/thaw cycles (liquid nitrogen/water bath, T > 50°C) were performed. Subsequently, 100 nm diameter large unilamellar vesicles (LUVs) were prepared by the extrusion method using Polycarbonate membranes (Nuclepore, Track-Etch Membrane) from Whatman Scheider & Schuell and a Mini-extruder from Avanti Polar Lipids, at a temperature above the gel/fluid transition temperature of all the individual lipids present in the mixtures (T > 50°C).

### 2.3 Absorbance and fluorescence measurements and data analysis

The absorption spectra were recorded with a Jasco V-560 spectrophotometer (Easton, MD, United States).

The steady-state fluorescence emission and excitation spectra and fluorescence anisotropy measurements were performed using the Fluorolog-3 v2.2 spectrometer (HORIBA; Villeneuve D’ascq, France) with double monochromators at excitation and emission and a Xenon 450 W lamp as a light source, unless stated otherwise. In all the experiments 1 cm × 0.4 cm Hellma^®^ semi-Micro, Suprasil^®^ quartz fluorescence cuvettes were used. Samples were excited along the 1 cm pathway, and the emission collected along the 0.4 cm pathway.

The steady-state anisotropy *< r>* was calculated according to Eq. 1 (Loura et al., 2003)

$$\langle r\rangle =\frac{\left(I_{VV}\;–\;G\times I_{VH}\right)}{\left(I_{VV}+2G\times I_{VH}\right)}$$ (1)

where *I*_*XY*_ represents the emission intensity reading with vertical (*V*) or horizontal (*H*) orientations of the excitation (*X*) and emission (*Y*) polarizers, and G is the ratio $\frac{I_{HV}}{I_{HH}}$ which accounts for the different sensitivity of the detector to horizontally and vertically polarized light. An adequate blank was subtracted from each intensity reading, and each set of four intensity components was measured seven times.

Time-resolved measurements were performed with Fluorohub v2.0 (HORIBA) coupled to the spectrofluorimeter. The nanoLEDs (N-280, N-320, N-370 with UGI filter and N-460) used for pulsed excitation were also from HORIBA.

The analysis of fluorescence decay curves, both intensity and anisotropy, was performed using the Time-Resolved Fluorescence Anisotropy Data Processor v.1.4 program (Minsk, Belarus). The quality of the decay fitting parameters was evaluated by the reduced χ^2^, the residuals and the auto-correlation of the residuals.

A normalized fluorescence intensity decay can be described by a sum of exponentials:

$$I\left(t\right)=\underset{i}{\sum }\alpha_{i}\;\exp\left(\frac{-t}{\tau_{i}}\right)$$ (2)

where *α*_*i*_ and *τ*_*i*_ are the normalized amplitude and lifetime of component *i*, respectively. The intensity-weighted mean fluorescence lifetimes are given by Eq. 3:

$$\langle \tau \rangle =\frac{\underset{i}{\sum }\alpha_{i}\tau_{i}^{2}}{\underset{i}{\sum }\alpha_{i}\tau_{i}}$$ (3)

and amplitude-weighted mean fluorescence lifetimes are given by Eq. 4:

$${\langle \tau \rangle }_{a}=\underset{i}{\sum }\alpha_{i}\tau_{i}$$ (4)

### 2.4 Determination of membrane/water partition coefficients

The partition coefficient (*K*_*p*_, Eq.5 (White et al., 1998)) was determined using two different approaches: using the intrinsic fluorescence of the compounds and *via* the fluorescence quenching of the di-4-ANEPPS probe.

Kp=nCLVLnCWVW (5)

where $n_{C_{i}}$ are numbers of moles of the compound in each phase *i* (*L*–lipid, *W*–water) and *V*_*i*_ are volumes of the corresponding phases.

#### 2.4.1 Intrinsic fluorescence of the compounds

The *K*_*p*_ values of **L1** and **L2** were determined by varying the concentration (0–3 mM) of lipid in suspensions of DOPC LUVs and with a constant concentration of compound in *buffer 1*.

The compounds were left to incubate for 1 h with the DOPC bilayers and then steady state fluorescence intensity for each sample, at the maximum excitation and emission wavelengths for each compound were measured. The data obtained were analyzed according to Eq. 6, where *I* is the fluorescence intensity at each concentration, *I*_*L*_ is the limit fluorescence intensity of the compound in the lipid (when all the compound is partitioned to the membrane), [*L*] is the concentration of the lipid, *I*_*W*_ is the fluorescence intensity of the compound in aqueous (buffer) solution, (*W*) is the molar water concentration in the lipid suspension, considered to be equal to that of pure water at 25°C (55.3 mol/L) (White et al., 1998; Loura et al., 2003).

$$I=\frac{I_{L}K_{p}\left[L\right]+I_{W}\left[W\right]}{\left[W\right]+K_{p}\left[L\right]}$$ (6)

The parameters *K*_*p*_ and *I*_*L*_ were determined by fitting Eq. 6 to the experimental data using a non-linear regression by the least squares method.

After determining the *K*_*p*_ it was possible to compute the compound mole fraction in the membrane, *x*_*C*_ for each lipid concentration:

$$x_{C}=\frac{K_{p}\left[L\right]}{\left[W\right]+K_{p}\left[L\right]}$$ (7)

#### 2.4.2 Di-4-ANEPPS fluorescence quenching

For determination of the *K*_*p*_ values of **1** and **2**, the lipid (DOPC) and probe (di-4-ANEPPS) concentrations remained constant, and the compounds concentrations were altered. The partition of a compound to the membrane was determined from the decrease of the fluorescence intensity of di-4-ANEPPS due to the successive increase of compound concentration in the membrane. LUVs were prepared in the Hepes buffer (without DMSO). The probe was added after extrusion and left overnight to incorporate. The next day, the LUVs were diluted and DMSO was added at 2% V:V. After that, the complexes in *buffer 2* were added. As a result, solutions with lipid concentrations of 0.2, 0.5, 1, 1.5 and 2.5 mM were obtained with the compounds **1** and **2** in the widest possible range of concentrations, limited essentially by their solubility (**1**: 0–22 μM; **2**: 0–24 µM). Samples were allowed to incubate for 1 h. The readings were performed on the SpectraMAX GeminiEM microplate reader (Molecular Devices).

Data showed the occurrence of two quenching processes; however, a better fitting was achieved when only the first points of the Stern–Volmer graphs were analyzed with a linear fit of Eq. 8 (de Castro et al., 2001):

$$\frac{I_{0}}{I}=1+K_{SV}\left[Q\right]$$ (8)

where *I*_*0*_ and *I* are the fluorescence intensity, respectively, in the absence and presence of compound, *K*_*SV*_ is the Stern–Volmer constant and [*Q*] is the concentration of quencher (in the present study, complexes **1** and **2**). In this case, the quencher is distributed between the membrane and the aqueous phase, and only the quencher molecules in the membrane will be effectively responsible for the fluorescence quenching.

Having:

$${\left[Q\right]}_{T}={\left[Q\right]}_{L}+{\left[Q\right]}_{W}$$ (9)

where *T*, *L* and *W* indexes represent total, lipid and water concentration of quencher, Eq. 8 can be rewritten as follows (de Castro et al., 2001):

$$\frac{I_{0}}{I}=1+K_{SV}^{ap}\times {\left[Q\right]}_{T}$$ (10)

in which $K_{SV}^{ap}$ is the apparent Stern–Volmer constant. This constant is retained through a linear or polynomial fit of the $\frac{I_{0}}{I}$ values for the various quencher concentrations. This value depends on the efficiency of the compound in decreasing the fluorescence of the probe, but also on its *K*_*p*_. The relationship between the $K_{SV}^{ap}$ and the *K*_*p*_ is given by Eq. 11:

$$K_{SV}^{ap}=K_{SV}\frac{K_{p}}{K_{p\;}\alpha_{L}+\left(1-\alpha_{L}\right)}$$ (11)

where $\alpha_{L}$ is the volume fraction of the lipid phase ( $\alpha_{L}$ = *V*_*L*_/*V*_*T*_) where in turn *V*_*L*_ is the volume of the lipid phase and *V*_*T*_ is the total volume. Assuming *V*_*L*_ << *V*_*T*_, Eq. 11 can be simplified to Eq. 12:

$$K_{SV}^{ap}=K_{SV}\frac{K_{p}}{K_{p\;}\alpha_{L}+1}$$ (12)

with the $K_{SV}^{ap}$ value known for each lipid concentration, the value of *K*_*p*_ can be retrieved by fitting Eq. 12 through a non-linear regression.

### 2.5 Study of the LUVs stability and permeability in the presence of the compounds

The stability of LUVs was evaluated by determining their size and surface charge in the presence and absence of the compounds. These measurements were performed for DOPC and for the DMPC:DMPG mixture (7:3 mol:mol). The first two lipids are zwitterionic and the mixture DMPC:DMPG is anionic (PG is negatively charged at physiological pH). Lipids were hydrated in an appropriate buffer to achieve the initial concentration of 2 mM. For this study we used *buffer 2*. After extrusion, the LUVs were stored overnight at 4°C. The next day, DMSO was added at 2% V:V. The concentration of the compounds in the samples was 20 μM. The compounds were incubated for 1 h with lipid bilayers before measurements. The experiments were conducted three times (about 15 runs each) with at least two replicates at 25°C.

The determination of the LUVs size distribution was performed at 25°C by dynamic light scattering (DLS) in a ZetaSizer Nano S. The zeta potential (*ζ*) (mV), related to the surface charge of the particles, was also measured using a ZetaSizer Nano Z by Laser Doppler Anemometry. (Corvo et al., 2015; Carreira et al., 2017). For each sample, a measurement (between 15 and 50 runs depending on the sample) was done at 25°C. Measurements were conducted for each system with at least two replicates.

The effect of the compounds on membrane permeability was evaluated by CF release (leakage) (Guillén et al., 2008, 2009) using a SpectraMAX GeminiEM microplate reader. The excitation and emission wavelengths were respectively 492 nm and 530 nm, with a cut-off filter at 515 nm.

The lipids were hydrated with *buffer 1* containing CF (40 mM). After extrusion, the non-encapsulated CF was separated from the suspended vesicles by gel filtration with a Sephadex G-75 column and elution with Hepes buffer 50 mM pH 7.4. DMSO (2% V:V) was added after the LUVs elution. After placing the LUV suspension in the microplate wells (final concentration of lipid 0.5 mM), the compounds were added at a final concentration of 10 μM. Fluorescence intensity measurements started immediately after the addition of the compounds and continued for 24 h with shaking between each fluorescence intensity reading.

After 24 h, Triton X-100 was added to all wells at a final concentration of 0.5% (w/V) and fluorescence intensity was measured to determine the maximum percentage of release of CF (*F*_*100*_). The CF leakage percentage was calculated using Eq. 13:

$$\%\;Leakage=\frac{F_{t}-F_{0}}{F_{100}-F_{0}}$$ (13)

where *F*_*t*_ is the fluorescence intensity value at each time point, *F*_*0*_ is the initial fluorescence intensity value and *F*_*100*_ is the fluorescence intensity value after the addition of Triton X-100 (Guillén et al., 2009). To describe the experimental curves of the leakage percentage over time, an exponential function with one or two components (Eq. 14) was applied (Guillén et al., 2008):

L=L11−exp−tτL1+L21−exp−tτL2 (14)

where $L_{1}$ and $L_{2}$ represent the maximum leakage associated with each kinetic constant, *t* is the time after the addition of the compounds, and $\tau_{L_{1}}$ and $\tau_{L_{2}}$ are the time constants. The average leakage time constant, <*τ*_*L*_>, can be calculated using Eq. 15 and Eq. 16 (Guillén et al., 2008):

$$<\tau_{L}>=a_{1}\tau_{L_{1}}+a_{2}\tau_{L_{2}}$$ (15)

$$a_{i}=\frac{L_{i}}{L_{\max}}$$ (16)

where *a*_*1*_ and *a*_*2*_ are the normalized fractional components of leakage and *L*_max_ = *L*_*1*_ + *L*_*2*_.

### 2.6 Effect of compounds on lipid bilayers through DPH and di-4-ANEPPS fluorescence

To study the effect of the compounds on the different lipid bilayers used throughout this study, two membrane probes were used, DPH and di-4-ANEPPS. LUVs with four distinct lipid compositions were prepared: DOPC, DPPC, POPC:POPE:SM:Chol in 1:1:1:1 mol ratio - the mixture that mimics the membrane of normal mammalian cells (**N-model**) and POPC:POPE:SM:Chol 36.2:23.6:6.8:33.4 mol ratio, the mixture that mimics the membrane of U-118 cancer cells (**C-model**) (Barceló-Coblijn et al., 2011; Khmelinskaia et al., 2014).

All the experiments were performed in *buffer 3* with constant concentration of the compounds (20 μM). The LUVs were prepared at 2 mM initial lipid concentration in the Hepes buffer (pH = 7.4), then the respective probe solution in methanol was added to obtain a probe: lipid molar ratio of 1:500, and the mixture was incubated for 1 h at a temperature higher than the transition temperature of the lipids. Methanol concentration was always kept below 1% (V/V). Then the mixture was stored overnight at 4°C in the dark. On the next day DMSO was added to have the same final concentration of 5% (V/V) in all samples analyzed. After addition of the stock solutions of the compounds, the mixtures were incubated for 1 h before the fluorescence measurements, which were also performed for the control and background samples. Fluorescence measurements were performed at room temperature (approximately 24°C).

For both probes, excitation and emission spectra, steady-state fluorescence anisotropy and fluorescence intensity decays were measured, and in addition, fluorescence anisotropy decays in the case of DPH only. The value of steady-state anisotropy calculated for DPH, through anisotropy decays, was compared with that of steady state measurements. In all cases the values were remarkably similar and, therefore, only the anisotropy values calculated through the parameters resulting from the anisotropy decays are presented.

Shortly, the fluorescence intensity decays measured in the horizontal and vertical polarization axes translate into anisotropy decays (Eq. 17, analogous to Eq. 1) which can be described by mono or multiexponential curves. The fluorescence anisotropy decay can be described by Eq. 18:

$$r\left(t\right)=\frac{\left(I_{VV}\left(t\right)\;–\;G\times I_{VH}\left(t\right)\right)}{\left(I_{VV}\left(t\right)+2\;G\times I_{VH}\left(t\right)\right)}$$ (17)

$$r\left(t\right)=\overset{n}{\underset{i=1}{\sum }}\beta_{i}\exp\left(\frac{-t}{\Phi_{i}}\right)+r_{\infty }$$ (18)

in which *n* is the number of components that contribute to the value of anisotropy in steady state, β_*i*_ is the fractional anisotropy for each rotational correlation time, *Φ*_*i*_, *t* is time, and *r*_*∞*_ is the limit value of anisotropy at infinite time. The factor G has the same meaning as for the steady-state fluorescence anisotropy (Eq. 1), and was determined as previously described (Bagulho et al., 2015; Starosta et al., 2020).

The sum of the fractional anisotropies with the limit value of the anisotropy decay gives the value of fundamental anisotropy of the fluorophore (*r*_*0*_; Eq.19):

$$r_{0}=\overset{n}{\underset{i=1}{\sum }}\beta_{i}+r_{\infty }$$ (19)

Fluorescence anisotropy decays also allow calculating the value of the steady-state anisotropy using Eq. 20:

$$\langle r\rangle =\frac{\int_{0}^{\infty }i\left(t\right)∙r\left(t\right)\;dt}{\int_{0}^{\infty }i\left(t\right)\;dt}$$ (20)

which for a fluorophore with three lifetime components and two rotational correlation times, like in the case of DPH, leads to Eq. 21:

$$\langle r\rangle =\frac{\beta_{1}\Phi_{1}\left(\frac{\alpha_{1}\tau_{1}}{\Phi_{1}{+\tau }_{1}}+\frac{\alpha_{2}\tau_{2}}{\Phi_{1}{+\tau }_{2}}+\frac{\alpha_{3}\tau_{3}}{\Phi_{1}{+\tau }_{3}}\right)+\beta_{2}\Phi_{2}\left(\frac{\alpha_{1}\tau_{1}}{\Phi_{2}{+\tau }_{1}}+\frac{\alpha_{2}\tau_{2}}{\Phi_{2}{+\tau }_{2}}+\frac{\alpha_{3}\tau_{3}}{\Phi_{2}{+\tau }_{3}}\right)}{\alpha_{1}\tau_{1}+\alpha_{2}\tau_{2}+\alpha_{3}\tau_{3}}+r_{\infty }$$ (21)

Both equations, Eq. 20 and Eq. 21, show that the steady-state anisotropy value is influenced by the average fluorescence lifetimes, so, for example, an increase in the value of anisotropy does not always mean an increase in system order, but may be the result of a decrease in the average fluorescence lifetime. Thus, it is essential to compare the value of <*r*> with the associated parameters (β_*i*_, *Φ*_*i*_, *r*_*∞*_) and, for this reason, the values of anisotropy in steady state discussed throughout this paper correspond to the value calculated by Eq. 21.

## 3 Results and discussion

**FIGURE 2:**
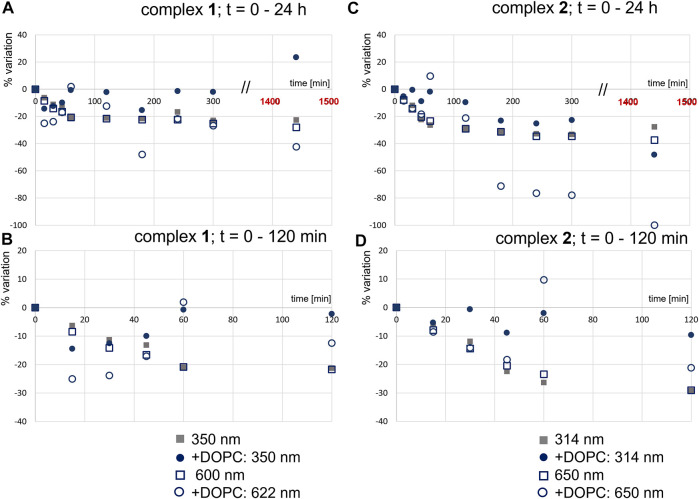
Effect of DOPC LUVs on the stability of **1 (A** and **B)** and **2 (C** and **D)**: variation in absorbance (%) at the indicated wavelengths, in the absence (c_**1**_ = 55 μM; c_**2**_ = 61 µM) and presence of DOPC LUVs (c_**1**_ = 25 μM; c_**2**_ = 27 µM) **(B)**. All samples were prepared in *buffer 2*; [DOPC] = 1.0 mM.

We determined the stability of the complexes in the buffered solutions with or without DOPC LUVs. To do so, we analyzed two bands in the absorption spectra of **1** (Figures 2A,B) and **2** (Figures 2C,D). Figure 2 shows the stability of the complexes from the spectra in the UV-Vis region measured during 24 h and corresponding absorption spectra are presented in Supplementary Figure S4, S5. The lowest energy band, in the visible region, is a ligand-to-metal charge-transfer band (O_phenolate_ - Ru^III^) (Matos et al., 2013), with maxima at 600 and 650 nm, respectively. The second band, with maxima around 350 nm for **1**, and around 314 nm for **2,** results from the transitions at the coordinated triphenylphosphane ligand. In *buffer 2*, one can observe a regular decrease in the intensity of these bands for both complexes, and clearly the changes are more pronounced for **2**. In the presence of DOPC LUVs, variations are more irregular, which is expected for such micro-heterogeneous systems.

### 3.1 Interaction of ligands and complexes with the membrane

For a first assessment of the interaction of compounds with lipid bilayers, DOPC LUVs were chosen, because this is a fluid lipid bilayer at room temperature. This lipid phase usually facilitates the incorporation of exogenously added compounds, the bilayers are very stable, and the samples have very low turbidity. Moreover, the absence of phase separation/lipid domains renders the meaning of the partition coefficients more straightforward (Loura et al., 2003).

#### 3.1.1 Intrinsic fluorescence of compounds in the absence and presence of DOPC LUVs

**FIGURE 3:**
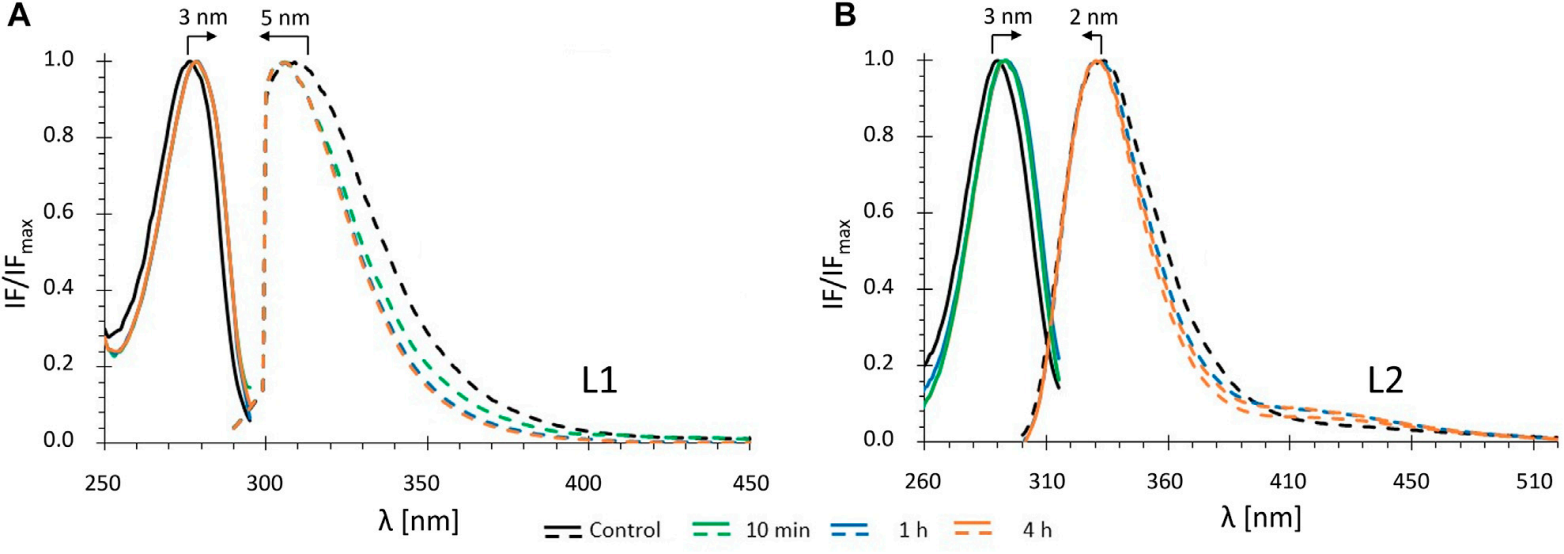
Fluorescence spectra of ligands **L1 (A)** and **L2 (B)** in *buffer 1*: normalized excitation (solid lines) and emission spectra (dashed lines) in the absence (control: black) and in the presence of DOPC LUVs ([DOPC] = 1.0 mM) for different incubation times: green - 10 min; blue - 1 h; orange - 4 h (c_**L1**_ = 20 μM; c_**L2**_ = 15 µM).

In this study we characterized the intrinsic fluorescence of the free *salan* ligands. The fluorescence of the ligands in **1** and **2** was virtually not observed. The fluorescence of each compound was characterized in buffered solution (as a control) and in the presence of DOPC LUVs at three incubation timepoints (10 min, 1 h and 4 h) with the liposomes. Figure 3 shows excitation and emission spectra of **L1** and **L2** in the presence and absence of lipid.

**FIGURE 4:**
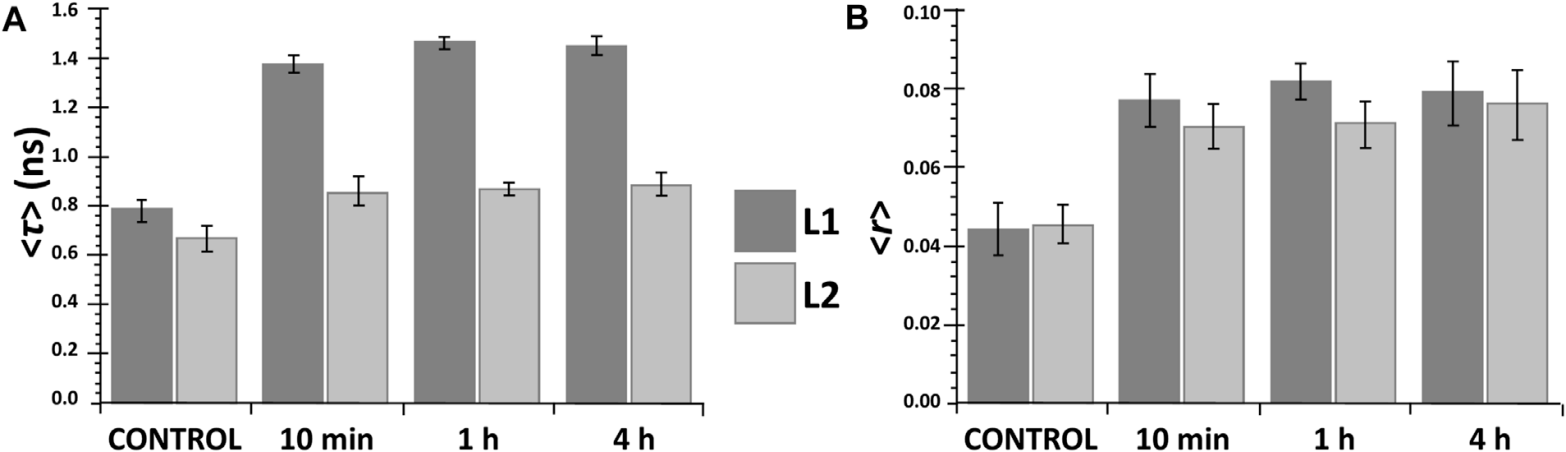
Mean fluorescence lifetime **<τ> (A)** and steady-state fluorescence anisotropy **<r> (B)** of the ligands **L1** (dark grey) and **L2** (light grey) in the absence (control) and presence of DOPC LUVs at different incubation times: (c_**L1**_ = 20 μM; c_**L2**_ = 15 µM).

**FIGURE 5:**
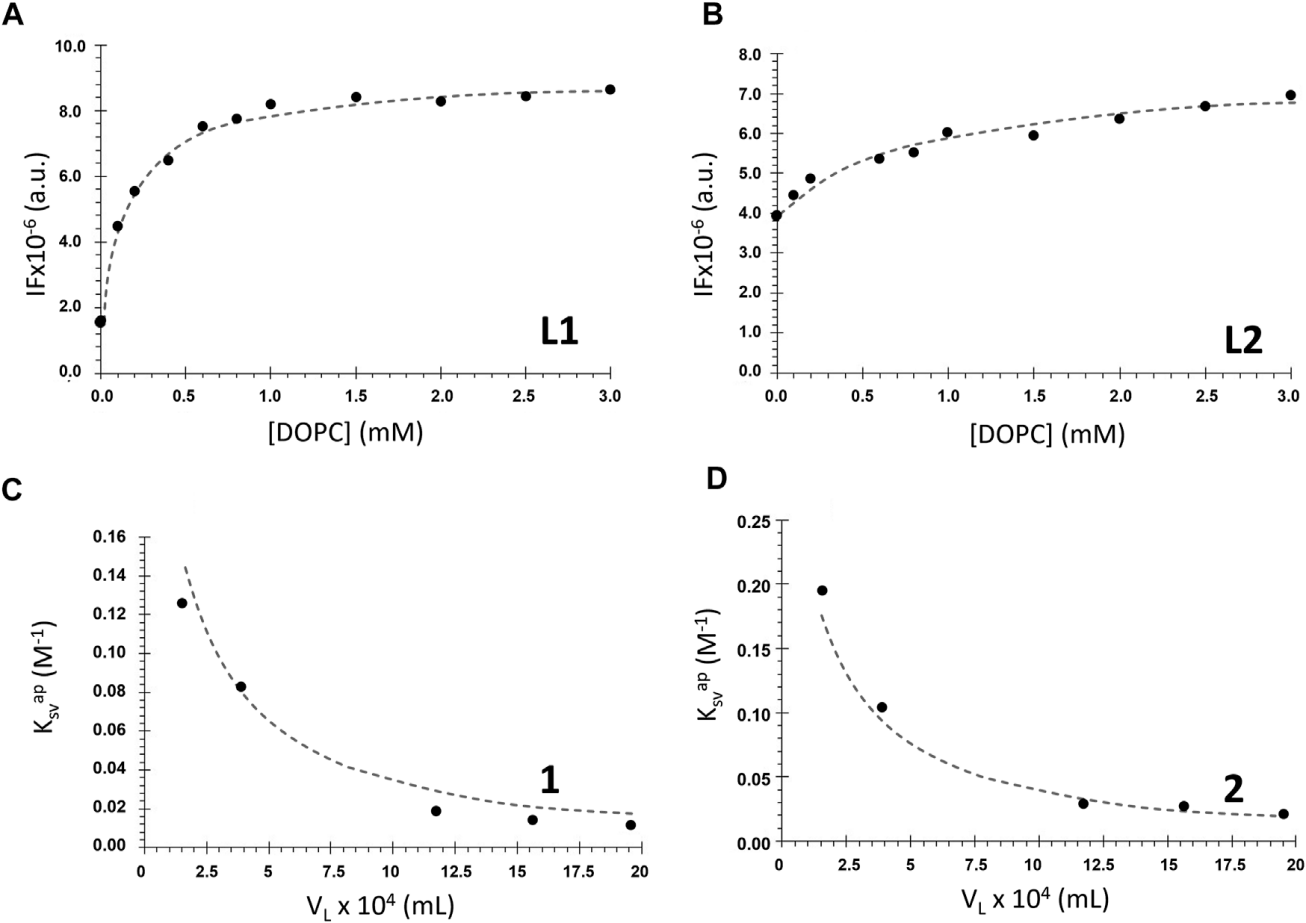
Membrane/water partition coefficient determination for the ligands **L1** and **L2** and complexes **1** and **2**. **(A)**, **(B)** Ligands fluorescence intensity (IF) in the presence of DOPC LUVs with increasing lipid concentrations (*buffer 1*, c_**L1**_ = 20 μM, *λ*_ex_ = 277 nm and *λ*_em_ = 304 nm; c_**L2**_ = 10 μM, *λ*_ex_ = 290 nm and *λ*_em_ = 340 nm). The dashed lines represent the non-linear fitting of Eq. 6 to the data. **(C)**, **(D)** Apparent Stern–Volmer constant values for the quenching of di-4-ANEPPS fluorescence by the complexes as a function of the lipid phase volume in 1 ml of LUVs suspension (*buffer 2*, c_**1**_: 0–22 μM; c_**2**_: 0–24 µM). The dashed line represents the non-linear fit of Eq. 12 to the data.

Analysis of the excitation and emission spectra shows that, for both ligands, binding to the DOPC LUVs results in small red shifts in excitation maxima and blue shifts in the emission maxima (Figures 3A,B), 5 nm for **L1** and 2 nm for **L2**. These shifts were observed after a relatively short incubation time (10 min) and did not change in time (at least up to 4 h). Also, both the average fluorescence lifetime and fluorescence anisotropy of **L1** and **L2** increased significantly in the presence of DOPC LUVs after 10 min (Figures 4A,B) and remained virtually constant till 4 h of the experiments. In general, the changes in the fluorescence properties were stronger for **L1** than for **L2.** This may result from different fractions of the compounds in the DOPC membrane, as will be discussed further in the next section. However, they can also be due to the fact, that both ligands, despite their structural similarity, are two distinct fluorophores. For both, the first absorption and emission bands are the result of a π→π* transition in the aromatic rings, but the presence of the two electron-donor hydroxyl and methoxy groups strongly affects the exact transition mechanisms (hence the red shift in absorption when going from a more polar to a less polar medium). In **L1** these groups are in *meta*, and in **L2** are in *para* positions, leading to different electron distribution around the phenyl rings.

#### 3.1.2 Membrane/water partition coefficient of the compounds

The membrane/water partition coefficient (*K*_*p*_, Eq. 5) allows to quantify the distribution of the compounds between the lipid bilayer and water. The *K*_*p*_ values of the tested compounds were determined using two approaches: 1) employing the intrinsic fluorescence of the compounds and 2) using the fluorescence of the probe di-4-ANEPPS.

Intrinsic fluorescence of *salan* ligands **L1** and **L2** (alone) was used successfully by fitting Eq. 6 to the data (Figures 5A,B). The virtual absence of emission of the coordinated ligands in **1** and **2** prompted us to study the partition of the complexes to the lipid bilayer through di-4-ANEPPS fluorescence quenching. It was observed that the complexes caused a decrease in the probe fluorescence intensity [data for [DOPC] = 1.5 mM is shown in Supplementary Figure S6], and this change was used to determine the *K*_*p*_ values for the complexes (Figures 5C,D), as described in Materials and Methods.

Table 1 contains the *K*_*p*_ values obtained for the tested compounds. It shows that the ligands are characterized by high and quite different values of *K*_*p*_. Most probably, this is a result of their different volume, which translates into more stereochemical hindrances to enter the membrane in the case of **L2**.

**TABLE 1:** Membrane/water partition coefficient (*K*_*p*_) values of the complexes and ligands for DOPC bilayers, calculated using Eq. 6 (**L1** and **L2**) or Eq. 12 (**1** and **2**) to the experimental data.

Compound	*K*_*p*_/10^4^	
	Intrinsic fluorescence	Di-4-ANEPPS fluorescence
**L1**	26.2 ± 1.6	
**L2**	5.97 ± 0.08	
**1**		0.8 ± 0.5
**2**		1.4 ± 0.5

*K*_*p*_ values for complexes **1** and **2** are one order of magnitude smaller than the values for the *salan* ligands. The weaker partition of the complexes may result from their significantly larger size, as well as their higher polarity. To interpret the differences between the ligands and the complexes, it is also necessary to consider that “partition” is not necessarily equal to “incorporation”. The compound can interact with the bilayer at the surface and, thus, stereochemical impediments may not constitute an explanation for the different *K*_*p*_ values. Moreover, this difference can also be justified by the coordination of the ligands to the metal center, with another ligand (PPh_3_) influencing their incorporation into the membrane. On another hand, the methoxy groups positions may have some influence on the interaction with the membrane, and if so, it is expected that complex **1** will present *K*_*p*_ slightly lower than that of **2**, since this group is in a stereochemically more impeded position, closer to the co-ligands (Figure 1). Nevertheless, results indicate that all the tested compounds have *K*_*p*_ values comparable to or higher than those of commercial membrane probes, such as di-4-ANEPPS which has membrane/water partition coefficients for fluid membranes in the order of 10^4^ (Bastos et al., 2012). Importantly, these *K*_p_ values show that all the compounds have a stronger preference for the membrane over water.

The values of *K*_*p*_ allowed to calculate the mole fraction of compound effectively present in the DOPC bilayer phase for each lipid concentration through Eq. 7 (see Supplementary Figure S7). For example, at a typical 1 mM lipid concentration, **L1** is approximately 80% partitioned to the membrane, whereas **L2** is less than 50%. This can explain the larger changes in the fluorescence properties observed for **L1** than for **L2**, as mentioned in the previous section. For the complexes **1** and **2**, the percentage in the membrane is only ca. 13% and 20%, respectively. Still, despite their much smaller partition to the membrane, the complexes are the ones having the greatest effects on the bilayer, as will be shown in the next sections.

### 3.2 Lipid bilayer permeability and stability

#### 3.2.1 Effect of compounds on membrane permeability

The effect of the compounds on membrane permeability was assessed by leakage of CF encapsulated in DOPC LUVs in the presence and absence of the compounds, as described under Materials and Methods. Table 2 shows the values of maximum leakage (*L*_max_) and the mean leakage time, <*τ*_*L*_ > calculated using Eqs 15, 16 after fitting Eq. 14 to the experimental curves. It should be noted that the results from the two controls (DOPC in buffer with and without DMSO) demonstrate that there is no noticeable release of the probe over time and that DMSO has no effect on membrane permeability as well. As such, differences in the percentage of leakage over time can be safely assigned to the effect of each compound. The data show that PPh_3_ is the compound inducing the largest and fastest leakage effect. *Salan* ligands have only a minor effect, with **L1** having a greater but slower consequence on permeability than **L2**. The complexes **1** and **2** (maximum leakage of 65%–70%) have a similar effect on the permeability of the bilayers. However, the increase in permeability caused by **2** is about 50% slower than the increase caused by **1**. Considering stability results, it is possible that molecules of the complexes in the membrane start decomposing after ca. 3h, and the effect is more pronounced in the case of **2**, which seems to have a stronger partition to the membrane. The slow but strong effect of the complexes on leakage can, therefore, be due to a partial release of the PPh_3_ ligand, which has a strong impact on the membrane permeability.

**TABLE 2:** DOPC membrane permeability parameters (*L*_max_ and <*τ*_L_>) obtained for each compound through CF leakage (Eqs 15, 16) after fitting Eq. 14 to the experimental results. (All values are the average ±standard deviation of at least 3 independent experiments).

Compound	*L*_max_	<*τ*_L_> (min)
**L1**	26 ± 1	356 ± 10
**L2**	15 ± 2	160 ± 9
PPh_3_	80 ± 1	35 ± 2
**1**	65 ± 4	161 ± 11
**2**	70 ± 6	264 ± 8

#### 3.2.2 Stability of LUVs in the presence of the complexes

Following the permeability results, an important aspect to be addressed was whether the compounds affect the lipid bilayer integrity and destabilize liposomal suspensions. To assess these effects, two membrane models were tested: DOPC and anionic DMPC:DMPG (7:3) LUVs, where the lipid DMPG provides a negative charge to the liposomes. This mixture was used to understand whether the presence of charged lipids affected the interaction of compounds with the membrane and in turn would affect the stability of the LUVs differently. Not only cell membranes possess some anionic lipids (Ciumac et al., 2019; Dubois and Jaillais, 2021), but also this mixture is widely used in drug delivery systems (Larabi et al., 2004; Gonçalves et al., 2017), because these liposomes are very stable and the negative charge prevents vesicles from aggregating and fusing (Howard and Levin, 2010; Rahnfeld et al., 2018). As such, the liposomes incubated with the compounds were characterized by dynamic light scattering, to obtain their size distribution, and zeta potential measurements (surface charge).

Table 3 shows the mean diameter values of the different LUVs in the absence and in the presence of compounds. Values between 106 and 120 nm were obtained for the mean LUV diameter, which is fully aligned with the size expected for LUVs formed by the extrusion method using a 100 nm pore diameter filter (Mayer et al., 1986).

**TABLE 3:** Mean diameter of LUVs composed by DOPC and DMPC:DMPG (7:3) and zeta potential (*ζ*) of DMPC:DMPG LUVs (7: 3) in the absence (controls) and presence of the compounds (1 h incubation, c = 20 μM, except c(PPh_3_) = 10 µM). Controls refer to samples without compounds.

	DOPC	DMPC:DMPG	DMPC:DMPG
	Mean diameter (nm)	Mean diameter (nm)	ζ (mV)
*buffer 1*			
Control	111.0 ± 0.4	101.1 ± 1.6	-23.9 ± 0.8
**L1**	111.1 ± 0.5	102.5 ± 0.3	-27.2 ± 0.3
**L2**	111.5 ± 0.2	102.2 ± 0.3	-24.2 ± 0.2
PPh_3_	111.5 ± 0.3	102.6 ± 0.4	-24.6 ± 1.4
*buffer 2*			
Control	112.5 ± 0.1	107.7 ± 1.6	-52.2 ± 4.3
**1**	114.7 ± 0.9	109.5 ± 0.8	-53.9 ± 4.4
**2**	113.8 ± 0.8	109.5 ± 1.5	-53.9 ± 4.0

For DOPC and DMPC:DMPG LUVs, it was observed that, when compared to the corresponding controls, there was no change (within experimental error) in their size upon incubation with the compounds. Accordingly, the maximum polydispersity index (PI) (Corvo et al., 2015) obtained for DOPC LUVs was 0.097 and for DMPC:DMPG LUVs 0.108, indicating that the size distribution range found is quite small.

The zeta potential values obtained for DMPC:DMPG LUVs in the absence and in the presence of compounds (the zeta potential value for DOPC LUVs is zero) are also presented in Table 3. Again, no differences in the liposomes zeta potential are found in the presence of any of the tested compounds when compared to the respective control. The large difference found between the control in *buffer 1* and *buffer 2* is due to the presence of NaCl in *buffer 1*.

The results obtained by dynamic light scattering and zeta potential measurements show that, even though the compounds are able to induce membrane permeabilization, they do so without compromising the LUVs integrity. This result is important regarding their mechanism of action, since alteration of plasma membrane permeability disrupts cellular homeostasis, which can ultimately lead to cell death. It is also a relevant result concerning the future development of liposome-based drug delivery systems for these compounds, as their incorporation in the liposomes does not affect their stability.

### 3.3 Biophysical studies with the probe DPH

To further study the effect of complexes **1** and **2** on lipid bilayers, we employed DPH, a fluorescent membrane probe that is located inside the membrane, in the less polar zone, parallel to the phospholipid acyl chains. The microenvironment of the probe which influences its fluorescence properties makes DPH a probe of choice as a first approach to study the organization of membranes (Lentz, 1989; Lentz, 1993).

We tested four model membranes. Two of them, consisting only of one single lipid, represent two extremes of membrane ordering, a more disordered phase (fluid phase) consisting of DOPC, and an ordered phase (gel phase) consisting of DPPC. The other two membrane compositions correspond to more complex model systems mimicking the plasma membranes of normal mammalian cells **N-model**: POPC:POPE:SM:Chol in molar proportion 1:1:1:1 and the U-118 cancer cells (**C-model**: POPC:POPE:SM:Chol in molar proportion 36.2:23.6:6.8:33.4) (Barceló-Coblijn et al., 2011; Martin et al., 2013; Khmelinskaia et al., 2014). For all the subsequent studies a Hepes buffer (pH = 7.4) with 5% DMSO (*buffer 3*) was used.

In initial experiments with **L1** and **L2** for DOPC LUVs it was observed that the presence of these ligands did not affect both the fluorescence anisotropy and the fluorescence intensity decay of DPH, proving, together with the previous results, that the ligands do not significantly affect the bilayer structure. Therefore, the experiments were performed only for the Ru(III) complexes, which were tested at a 20 μM concentration, in the range of IC_50_ values found for the complexes against MDAMB231 cells (Matos et al., 2013). All numerical data discussed is collected in Supplementary Table S1, and raw data is presented in Supplementary Figure S8, S9.

Importantly, the complexes tested in this work did not change the excitation and emission maxima wavelengths of the probe, regardless of the membrane composition used (see exemplary spectra in Supplementary Figure S8). This shows that the complexes are not interacting directly with the probe and that there is no dramatic reorganization of the lipids at the membrane core, in agreement with the dynamic light scattering results.

#### 3.3.1 Effect of the complexes on DOPC fluid bilayers

**FIGURE 6:**
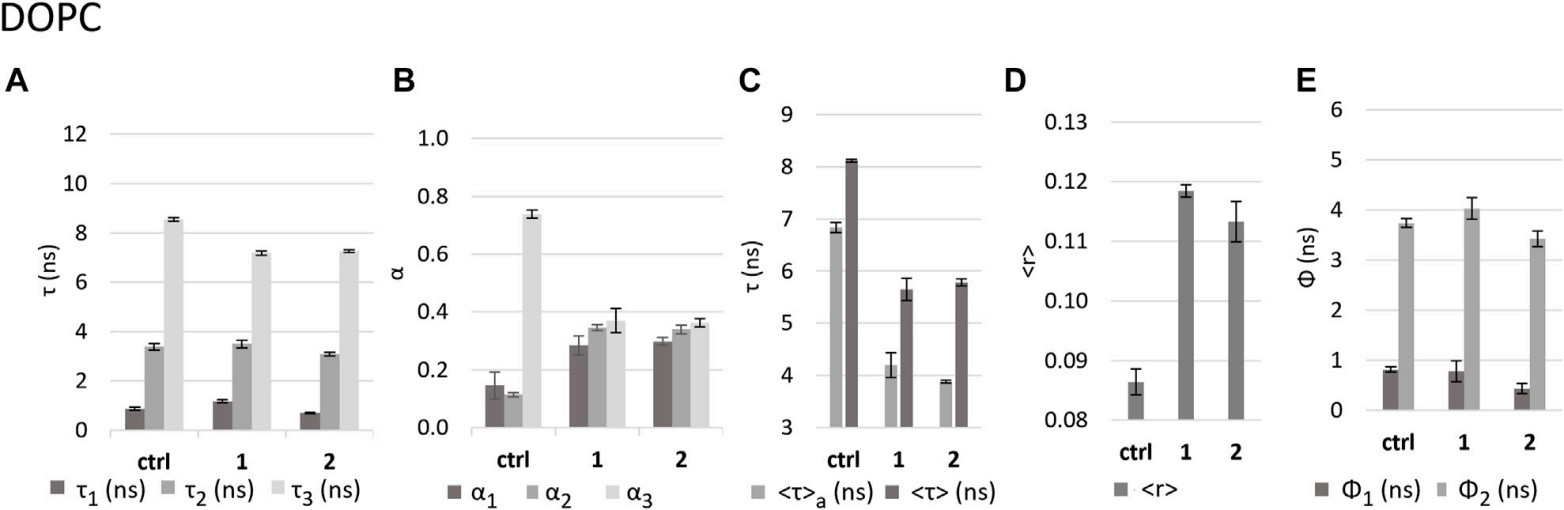
Effect of the complexes on DOPC LUVs as reported by DPH fluorescence properties. Values in the absence (control - ctrl) and presence of complexes **1** and **2** in *buffer* 3 of **(A)** fluorescence decay lifetime components *τ*_1_, *τ*_2_, and *τ*_3_; **(B)** amplitudes associated with each component; **(C)** intensity-weighted mean fluorescence lifetime (<τ>) and amplitude-weighted mean fluorescence (<τ>_a_) lifetime; **(D)** calculated fluorescence anisotropy in steady state <r>; **(E)** rotational correlation times Ф_1_ and Ф_2_.

DOPC LUVs allow us to observe the effect of **1** and **2** on a fluid membrane, which is in the liquid disordered phase at room temperature. We measured the fluorescence intensity decays of DPH in DOPC LUVs in the absence (control) and in the presence of the compounds. For the control, the fluorescence decay could be successfully described by the sum of two exponentials, having lifetimes of ca. 3 ns (medium component) and 9 ns (long component), which are typical for DPH labelling fluid membranes (Stubbs et al., 1981; Duportail and Weinreb, 1983). A third (short) component with a short lifetime of ca. 1 ns, contributing less than 2% to the total emitted light (Figures 6A,B), could be observed probably due to a high degree of hydration of this highly disordered lipid bilayer.

In the presence of the complexes, the fraction of light emitted by this short lifetime component increases to ca. 8% and 5% for **1** and **2**, respectively. The same is observed for the light fraction of the medium component (ca. 3 ns) which increases from ca. 6% to 29%–27% (Supplementary Table S1). Finally, the long component becomes shorter (ca. 7 ns). All these alterations result in a significant shortening of both *<τ>*_*a*_–the amplitude-weighted (Eq. 4) and *<τ>* - the intensity-weighted (Eq. 3) mean fluorescence lifetimes (Figure 6C) for both complexes.

The fact that a short component was required to describe the fluorescence intensity decay of DPH in the presence of the Ru complexes, brings important information about the compounds impact on the membrane. It should be noted that the short lifetime components of the DPH fluorescence decay originate from the fluorophore population located closer to the membrane-water interface (Konopasek et al., 1998) and an increase in this population indicates a shortened distance between DPH and water molecules (Konopasek et al., 2004). This strongly suggests that the complexes are causing an increase in the membrane hydration (Khmelinskaia et al., 2014), which is probably related to an increase in membrane permeability as shown above. The latter effect can be explained by an increase in polarity in the membrane environment through membrane hydration, because in polar media (such as water) the fluorescence quantum yield of DPH is very low (Gratton and Parasassi, 1995; Ho et al., 1995; Stubbs et al., 1995; Lakowicz, 2006; Guillén et al., 2009). This hypothesis is also supported by the observations of the decrease in di-4-ANEPPS fluorescence lifetimes in the presence of the complexes, which will be described in the next section.

The presence of the complexes causes an increase in DPH steady state fluorescence anisotropy (*<r>*) (Figure 6D). However, this increase can be explained by the fact that the complexes induce faster fluorescence intensity decays, and it is not necessarily caused by an increase in the order of the lipid acyl chains. Therefore, to fully understand the effect of the complexes on the fluidity of DOPC bilayers, we acquired fluorescence anisotropy decays. Analysis of the decays showed that DPH has two rotational correlation times, a shorter one of 0.8 ns and a longer one of ca. 4 ns. While **1** did not significantly change these values, **2** induced a significant decrease of both rotational correlation times (Figure 6E), which may suggest an increased fluidity, which is expected for a more strongly hydrated bilayer, in accordance with the smaller value of the short lifetime component and larger amplitude for **2** as compared to **1**.

#### 3.3.2 Effect of the complexes on DPPC gel bilayers

**FIGURE 7:**
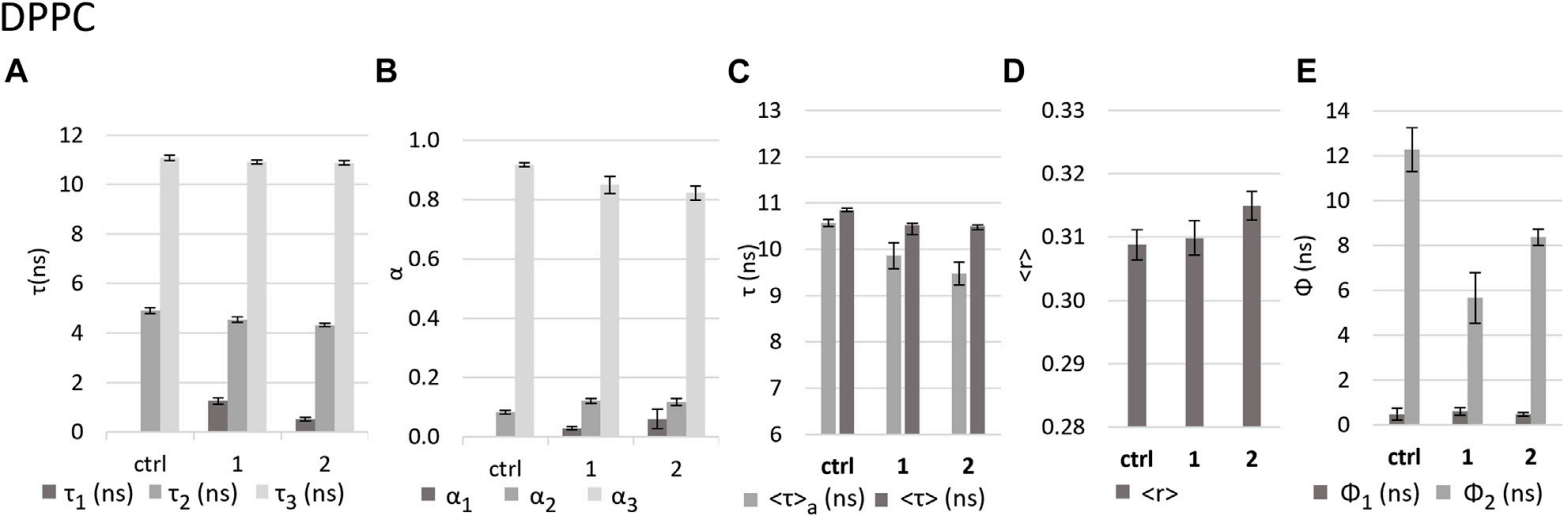
Effect of the complexes on DPPC LUVs as reported by DPH fluorescence properties. Values in the absence (control - ctrl) and presence of complexes **1** and **2** in *buffer* 3 of **(A)** fluorescence decay lifetime components; **(B)** amplitudes associated with each component; **(C)** intensity-weighted mean fluorescence lifetime (<τ>) and amplitude-weighted mean fluorescence (<τ>_a_) lifetime. **(D)** calculated fluorescence anisotropy in steady state <r>; **(E)** rotational correlation times Ф_1_ and Ф_2_.

DPPC at room temperature forms bilayers in the gel phase (Mabrey and Sturtevant, 1976; Davis, 1979), which translates into a lower hydration of the membrane, a tighter packing and slower diffusion of the lipid molecules and, consequently, also a lower rotational freedom of the probe DPH in the lipid bilayer. In the control, the fluorescence intensity decay of DPH was described by two components with lifetimes ca. 4.9 ns and 11 ns (Figure 7A), much longer than those found for DOPC, which is a consequence of the nature of the DPPC gel phase. Regarding the fluorescence anisotropy decays (and the steady-state anisotropy), the differences are even more marked. The long rotational correlation time shows a significant increase from ca. 4 ns in DOPC to ca. 12 ns in DPPC (Figure 6E and Figure 7E). Moreover, the limiting anisotropy of the probe, which was absent in DOPC, has now a high value of 0.3 (Supplementary Table S1: *r*_∞_), confirming that the rotation of the probe in the gel phase is both slower and much more hindered.

No notable alterations of steady-state fluorescence anisotropy, *<r>*, were observed in the presence of either **1** or **2** (Figure 7D). Regarding the time-resolved fluorescence anisotropy, however, the long rotational correlation time decreases by 64% in the case of **1** and 32% in the case of **2** (Figure 7E). This suggests that the impact on membrane fluidity is stronger for **1** than for **2**, contrary to the observation for DOPC.

The decrease in the *<τ>*_*a*_ values in the presence of the complexes (Figure 7C), by 7% and 10% for **1** and **2**, respectively, is about 3 times smaller than that observed for DOPC. This weaker effect on DPPC bilayers may be related to the fact that this lipid forms a more rigid and compact membrane that can hinder the entry of the complexes and thus minimize their effect. However, in the decay of fluorescence intensity (Supplementary Figure S9), the complexes induce the appearance of a very short lifetime component, with similar lifetime values as in the case of DOPC, (ca. 0.5 ns for **2** and ca. 1.3 ns for **1,** Figure 7A and Figure 6A), but much smaller amplitudes (Figure 7B and Figure 6B), since the control itself does not have short component at all, as expected for this less hydrated bilayer. The decrease in the average fluorescence lifetime is associated also with a small decrease in the *τ*_2_ and *τ*_3_ values and increase in the contribution of the medium component (*τ*_2_) with a concomitant decrease in the long component (*τ*_3_), similarly for both compounds. These results are compatible with an increase in polarity in the membrane environment, as in the case of DOPC, since water molecules tend to fill the appearing defects in the packing of membrane lipids (Ho et al., 1995).

#### 3.3.3 Effect of the complexes on lipid bilayers mimicking mammalian cell membranes

After analyzing the two extremes of disorder/order in the lipid bilayers, it was important to evaluate the effect of the complexes on bilayers that mimic more closely the composition of natural biological membranes. Two types of LUVs were used: **N-model** (POPC:POPE:SM:Chol 1:1:1:1) and **C-model** (POPC:POPE:SM:Chol 36.2:23.6:6.8:33.4). Importantly, in both mixtures, the coexistence of the liquid-disordered phase (*L*_*d*_) and the liquid-ordered phase (*L*_*o*_) can be observed (Ibarguren et al., 2013; Khmelinskaia et al., 2014), which mimics the presence of the membrane domains known as lipid rafts in mammalian cell membranes, and results from the presence of cholesterol and sphingomyelin (Sezgin et al., 2017). Compared to **N-model**, the **C-model** has a higher percentage of cholesterol and POPC, and a smaller amount of sphingomyelin. It is also known that cancer cells can have a higher percentage of cholesterol than normal cells, and that a decrease of its level can cause apoptosis of cancer cells, but also an increased invasion capacity in metastatic cells (Li et al., 2006; Maja et al., 2022). Therefore, the studies on the interactions of biologically active compounds with these two models is relevant for the development of therapeutics characterized by a more selective passive diffusion to the cell and/or to assess if the mechanism of action of the compounds may involve a reorganization of membrane domains (Kell and Oliver, 2014; Sharifian Gh, 2021; Vona et al., 2021; Maja et al., 2022).

**FIGURE 8:**
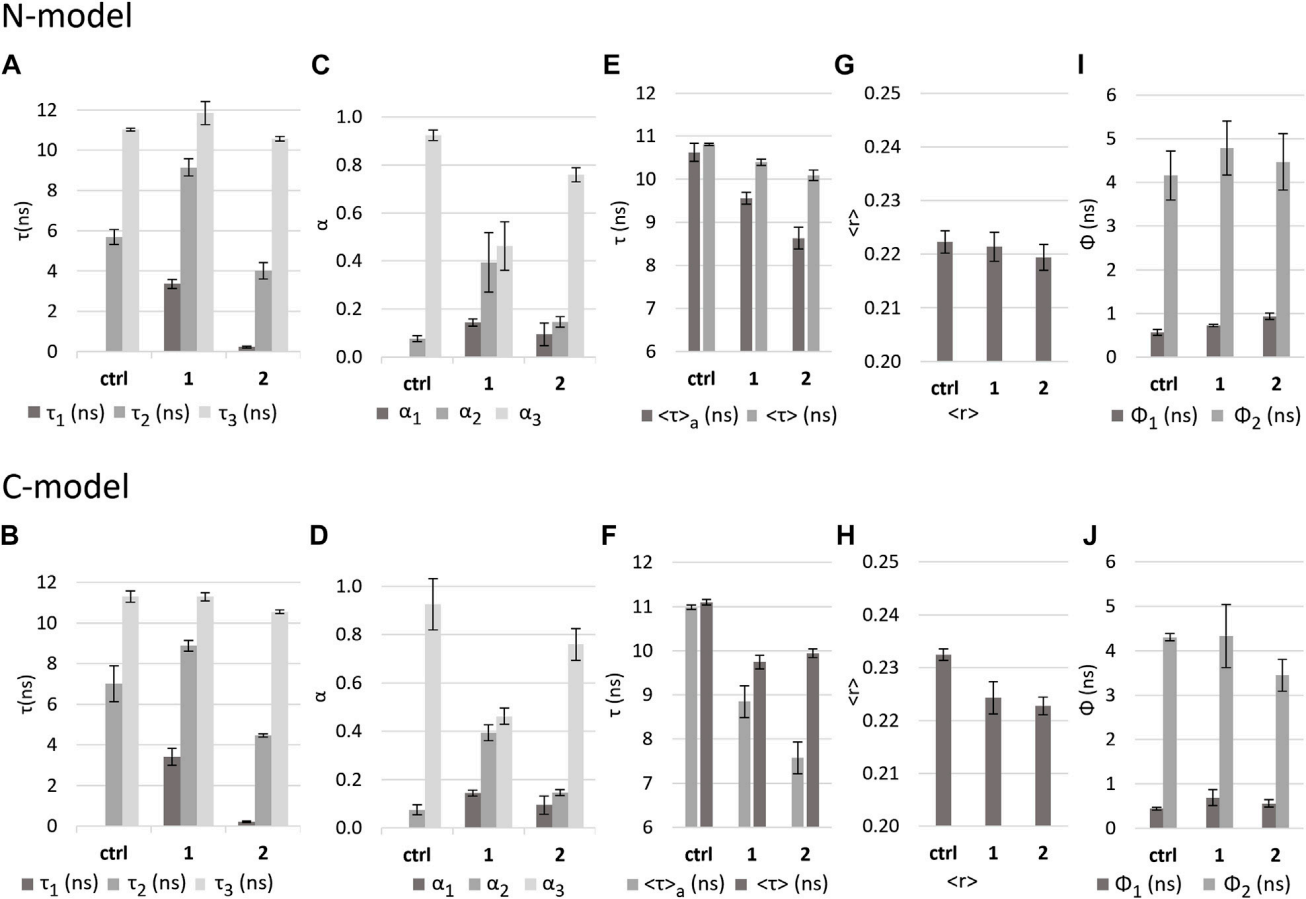
Effect of the complexes on **N-model** and **C-model** as reported by DPH fluorescence properties. Values in the absence (control - ctrl) and presence of complexes **1** and **2** in *buffer* 3 of **(A,B)**: fluorescence decay lifetime components; **(C,D)**: amplitudes associated with each component; **(E,F)**: intensity-weighted mean fluorescence lifetime (<τ>) and amplitude-weighted mean fluorescence (<τ>_a_) lifetime. **(G,H)**: calculated fluorescence anisotropy in steady state <r>; **(I,J)**: rotational correlation times Ф_1_ and Ф_2_.

For both models, the fluorescence intensity decay of DPH exhibits greater similarity to the values obtained for DPPC than for DOPC (see Supplementary Figure S9). However, the fluorescence anisotropy decay presents a long rotational correlation time that is closer to DOPC (Figures 8I,J) and a limiting anisotropy (ca. 0.2, Supplementary Table S1: *r*_∞_), which is between the one found for each pure lipid system. This behavior, intermediate between a gel and a pure *L*_*d*_ membrane, is typical of the *L*_*o*_ phase. Thus, it is also the expected one for lipid bilayers containing a significant fraction of *L*_*o*_ phase, in coexistence with a *L*_*d*_ phase, such as the **N-model** and **C-model** used in this work. There are also subtle differences between these two models. The medium lifetime component (*τ*_2_) is higher for the **N-model** (ca. 7 ns, Figure 8A) than for the **C-model** (ca. 5.7 ns, Figure 8B). Also, the short rotational correlation time is ca. 0.4 ns for the **N-model** and 0.6 ns for the **C-model**.

In the **N-model**, the presence of complexes **1** and **2** causes a *<τ>*_*a*_ decrease of 19% and 31%, respectively (Figure 8E). For the **C-model**, in the presence of complexes, a decrease in *<τ>*_*a*_ of 10% and 19% is observed (Figure 8F). As presented for the DOPC and DPPC bilayers, this decrease in lifetime can be explained by the appearance of a short component (Figures 8A,B) and a decrease in the contribution of the long component. Average lifetimes suggest that **2** has a greater impact than **1**, but the decrease in the amplitude of the long component (Figures 8C,D), assisted by the increase in the medium component is much more pronounced in the case of **1**. This was not the case with DOPC and DPPC, where the changes in the amplitudes of those components were similar for both complexes. Also, the short component that appears in the presence of the complexes is shorter than the one found for DOPC and DPPC in the case of **2** (ca. 0.2 ns), whereas it is much longer in the case of **1** (ca. 3.4 ns). Moreover, **1** caused a significant increase in the medium component lifetime value in **N-model** and **C-model** (from ca. 7 ns and 6 ns in the controls to ca. 9 ns), a distinguishing behavior from all the other situations.

These results suggest that the impact of each complex in the **N-model** and **C-model** might be qualitatively different. For **2**, the very short component of 0.2 ns is probably due to an increase in membrane hydration. However, the lifetime components obtained in the presence of **1**, suggest that instead, a membrane domain reorganization might be taking place. The short component and the increased medium component are more similar to a DOPC membrane than in the control, whereas the long component shows a slight increase from 11 to 12 ns solely in the **C-model**. The most straightforward explanation for these results is that the disordered phase is becoming more disordered, and the ordered phase is becoming more ordered, i.e., enriched in sphingomyelin and/or cholesterol.

The value of the DPH steady-state anisotropy for both models does not show any significant change in the presence of either of the complexes (Figures 8G,H). Nonetheless, there were significant changes in the fluorescence anisotropy decays. In both models, for compound **1**, the short rotational correlation time becomes longer and more similar to the one found in DOPC membranes, but no significant changes could be detected in the long component (Figures 8I,J). For compound **2**, the extent of the effects is different in each model. For the **N-model**, the short rotational correlation time increases slightly, also becoming closer to that of DOPC, and the long rotational correlation time decreases significantly, to a value much smaller than for compound **1**. Regarding the **C-model**, there is only a significant change in the short rotational correlation time, which increases to a value that is also closer to DOPC.

Considering these results and the changes described above in the fluorescence lifetimes, it seems that **2** leads in general to a fluidification of the membrane with an increased water penetration. with a larger impact on the **N-model**. Considering that **C-model** has a larger cholesterol fraction and is slightly less fluid than the **N-model**, this is in line with what was observed for DOPC and DPPC. In sum, the effect of **2** is to a large extent defined by the global fluidity of the membrane.

The results for **1** suggest a different scenario. This complex has a unique behavior when interacting with the complex lipid mixtures of the **N-model** and **C-model**, concerning both the fluorescence intensity and the fluorescence anisotropy decay. A possible explanation is that this complex exhibits some preference for the interface between *L*_*d*_ and *L*_*o*_ domains. Domain interfaces have a structure that differs from the bulk phase and thus, the complex could accommodate in those regions without inducing a strong perturbation, which would lead, for example, to an increased hydration. However, the changes of domain interface properties could result in a different mixing behavior of the lipids, changing the *L*_*d*_ and *L*_*o*_ phase composition/abundance that could explain the trend observed in both the fluorescence intensity and anisotropy decay of DPH in the presence of **1**.

These results show that the impact of the compounds can be highly dependent on the complexity of the lipid composition and phase behavior of the membrane, and in more complex systems the two compounds no longer have the same behavior, despite their apparent structural resemblances.

### 3.4 Biophysical studies using the probe di-4-ANEPPS

The fluorescence properties of DPH allowed us to demonstrate different effects caused by the Ru(III) complexes on the lipid bilayers; however, the results obtained showed only subtle differences in the interactions of the complexes between the mixtures that mimic cancer and normal cells. Thus, to further test the impact of **1** and **2** in **N-model** *versus* **C-model**, we used di-4-ANEPPS, which is located more at the surface of the membrane and is known for its ability to report membrane dipole potential (Montana et al., 1989) which can be modulated by the sphingolipid/cholesterol interactions in *L*_*o*_ domains/lipid rafts. In addition to being sensitive to the presence of sterols, di-4-ANEPPS has already been shown to have a greater partition for *L*_*o*_ phases (Bastos et al., 2012; Bandari et al., 2014), so it should allow us to study in more detail the effect of the complexes on *L*_*o*_ domains.

**FIGURE 9:**
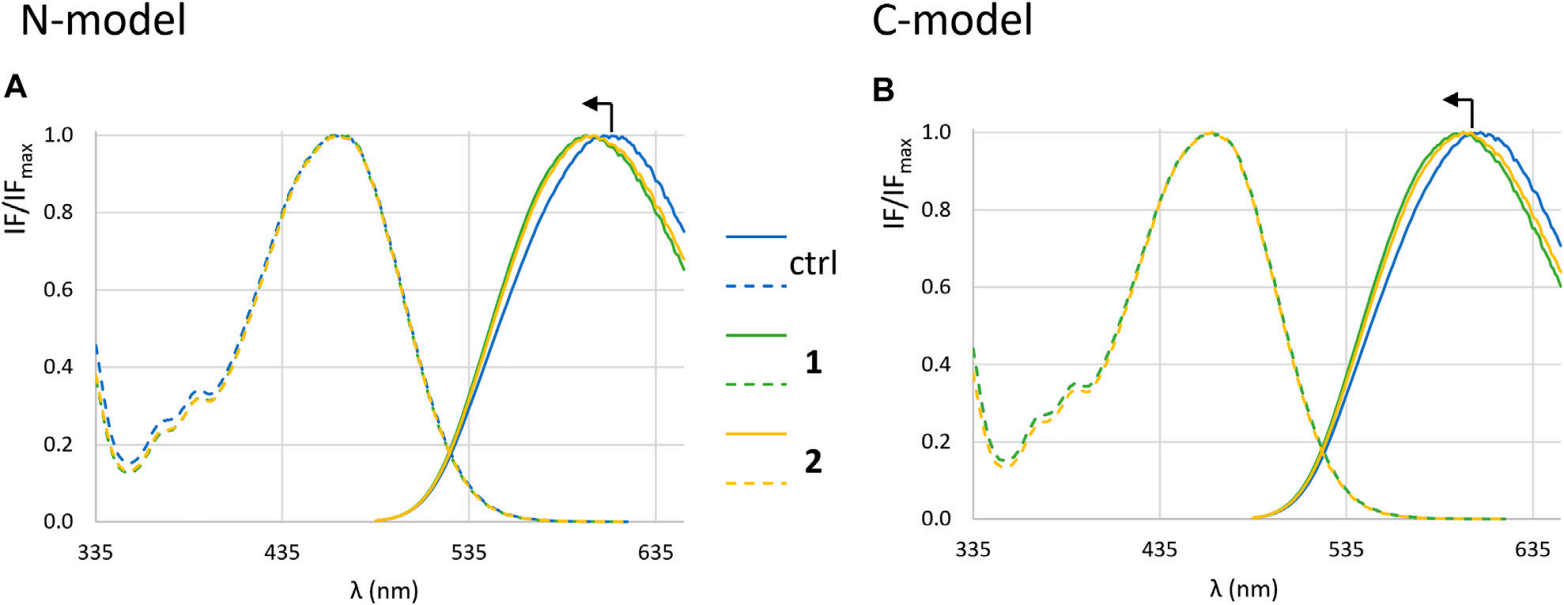
Fluorescence spectra of di-4-ANEPPS. Normalized excitation (dashed lines) and emission spectra (full lines) in the absence (blue) and in the presence of **1** (green) and **2** (yellow) in LUVs corresponding to **N-model (A)** and **C-model (B)** after 1 h incubation in *buffer 3*.

**FIGURE 10:**
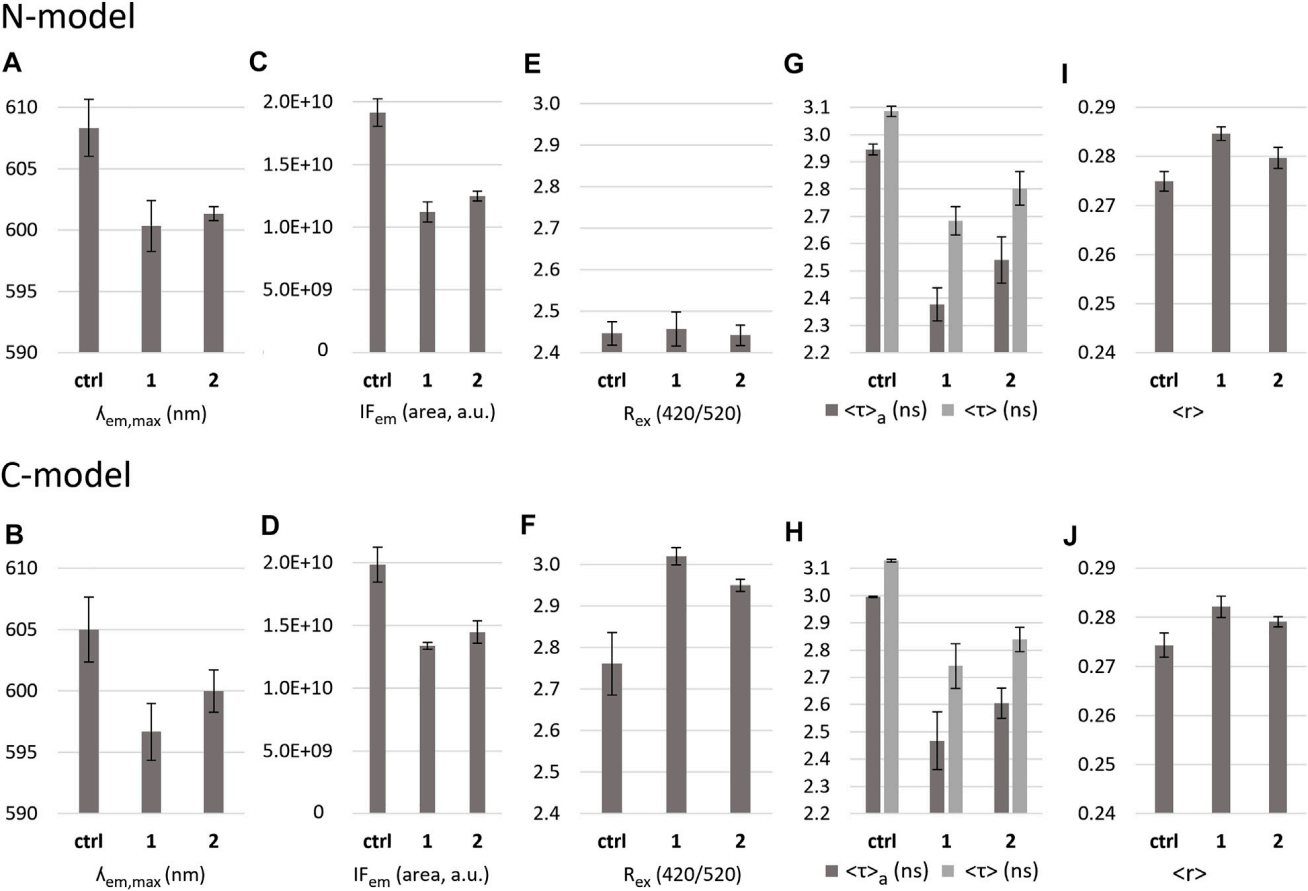
Effect of the complexes on **N-model** and **C-model** as reported by di-4-ANEPPS fluorescence properties. **(A,B)**: maximum emission wavelength (*?*_em,max_); **(C,D)**: fluorescence intensity measured as the area of the emission spectrum (IF_em_); **(E,F)**: average dipole potential measured as the ration of intensity at 420 nm and 520 nm excitation (R_ex_ (420/520)); **(G,H)**: average amplitude-weighted lifetimes <τ>_a_, and average intensity-weighted lifetimes <τ>, and **(I,J)**: fluorescence anisotropy <r> of the probe in the absence and the presence of complexes **1** and **2** in *buffer* 3.

Figure 9 shows the normalized excitation and emission spectra of di-4-ANEPPS, incorporated into LUVs with the two membrane compositions studied, in the presence and absence of complexes. For both complexes **1** and **2** no obvious deviations in the excitation spectra were observed, but the emission spectrum is blue-shifted ca. 8 nm in the presence of the complexes (Figures 10A,B). This suggests that the complexes are located close to the membrane surface, near the water-phospholipid headgroup interface (Bastos et al., 2012). As for the amplitude-weighted mean fluorescence lifetime of di-4-ANEPPS (Figures 10G,H) there is always a decrease in the presence of complexes, with complex **1** having the greatest effect on this parameter.

The ratiometric measurement that reflects the membrane dipole potential (R_ex_), determined as the ratio between intensity values at 420 nm and 520 nm of the excitation spectrum, allows to detect changes not observable by a simple inspection of the spectra, because small changes in the spectra are amplified in the ratio. For the **C-model** (Figure 10F) the value of R_ex_ is higher than the one measured for the **N-model** (Figure 10E), due to the higher molar fraction of cholesterol in the cancer cells mixture, leading to an increase in the dipole potential of the membrane (Khmelinskaia et al., 2014; Bandari et al., 2014). In the **N-model**, no variations of dipole potential are observed upon addition of the complexes. In the **C-model**, however, there is an increase in the presence of both **1** and **2**, being slightly stronger for **1**. An increase in R_ex_ could be due to the *L*_*o*_ phase becoming even more enriched in sphingomyelin and cholesterol, and a concomitant shift of POPC and/or POPE to the *L*_*d*_ phase, but could also result from a different H-bonding pattern and orientation of phospholipids headgroup and water molecules.

Because di-4-ANEPPS has a different transversal location and is preferentially located in the *L*_*o*_ phase, it will be much more sensitive to alterations in the *L*_*o*_ domains than DPH. These changes detected by di-4-ANEPPS, which are smaller for **2** than for **1**, could remain undetected by DPH. In the additional study conducted with di-4-ANEPPS in DOPC membranes (Supplementary Figure S11) the same trend was observed, that is, the **1** and **2** complexes promoted the increase in dipole potential: however, this time, **2** had a stronger effect than **1**, which was almost non-significant. This result suggests that the increase in dipole potential induced by **2** might be caused by a different mechanism than **1**, since neither there are *L*_*o*_ domains in the DOPC lipid system, nor membrane dipole potential enhancers as cholesterol. On another hand, membrane disordering *per se* would most probably decrease membrane dipole potential. Nevertheless, an alteration of the hydration layer, as suggested by the increased membrane permeability and water penetration in DOPC bilayers, can contribute to the observed increase in dipole potential. This could also affect the orientation of phospholipid headgroups. Another plausible hypothesis is that the dipole moment of the compound itself could be aligned in an orientation approximately parallel with the electric field that is generated by the membrane dipole potential. This would mean that the orientation and/or membrane penetration of the two compounds could be slightly different and also depend on membrane lipid composition. In the more complex mixtures, **N-model** and **C-model**, it is possible that both phenomena are occurring, but the orientation effect would be more relevant for **2** (affecting more strongly DOPC and DPPC), whereas the alteration of lipid rafts and membrane organization would be more decisive in the case of **1** (affecting more strongly **C-model** and **N-model**).

The results obtained with the probe di-4-ANEPPS in the **N-model** and **C-model** agree with those obtained using DOPC LUVs and for DPH. The global trend of DPH time-resolved data indicates a similar effect of **1** on both **N-model** and **C-model**. However, an increase, albeit small, in the value of the long lifetime component of DPH was observed in the **C-model** only, which is consistent with the di-4-ANEPPS dipole potential measurement (effect on the **C-model**, only). For **2**, we could only conclude a fluidification of the membrane from DPH, with some parameters becoming closer to those obtained for DOPC. The decrease in the mean fluorescence lifetime of di-4-ANEPPS and DPH are both consistent with an increase in bilayer hydration. However, the results with DPH show that this increase is noticed in the more hydrophobic region of the bilayer probed by DPH only for **2**. One is tempted to suggest that **2** has a more buried location in the membrane, which would result in more pronounced effect on DOPC and DPPC membranes, and **1** a more superficial location affecting the H-bonding network at the membrane surface that stabilizes *L*_*o*_ domains, contrary to previous observations of phenolic acids (Filipe et al., 2018), which partition preferentially for disordered membranes, and destabilize cholesterol-rich domains, while lowering the membrane dipole potential. Spectral deviations and increased dipole potential of the membrane are consistent with an alteration of the dielectric properties of the membrane surface, particularly in the mixture that mimics the membrane of cancer cells.

## 4 Conclusion

In the present study, we have addressed the relevance of drug-membrane interactions in the context of the development of metal complexes as a promising strategy for cancer therapy. Characterizing these interactions is crucial to understand the mechanisms of transport of compounds into the cell, and evaluate the plasma membrane as a potential target, where the compounds may exert meaningful effects regarding their biological action. In this work we used two Ru(III) complexes with similar structures, differing only by the position of the methoxy groups in the benzene rings of coordinated *salan* ligands. We were particularly focused on assessing whether changes in membrane lipid composition and biophysical properties could lead to a different interplay with the compounds. For the initial studies we used two simple model systems on the opposite edges of the fluidity scale, and then we moved to more complex systems.

Changes in the biophysical properties of the membrane were observed in the presence of the complexes, in all types of membrane studied. Importantly, both complexes increased the leakiness of the DOPC membrane without compromising its integrity. As a first conclusion, these results indicate that the plasma membrane lipids can be a target for this kind of compounds, and that drug-membrane interactions should be considered when southing to unravel their mechanism of action and make progress in drug development.

In the two simple lipid systems, one highly fluid and disordered, and the other ordered and rigid, the compounds had qualitatively similar effects, namely a fluidization and increased hydration of the membrane. However, the most interesting results were obtained for the complex systems mimicking mammalian cell membranes, with slightly different compositions, reflecting the known differences between a healthy cell line and a pathological counterpart. These membranes do not differ dramatically in their biophysical properties, but they have a small difference in the fraction and composition of the *L*_*o*_ and *L*_*d*_ domains, one being slightly more ordered than the other. In these two systems, the addition of the complexes, despite their structural similarity, had qualitatively different outcomes on the membrane. This highlights the importance of the complexity of biological membranes, and their specific lipid composition and biophysical properties for the action of potential drug candidates. The relevance of these results becomes more evident when invoking that membrane lipid composition is altered in many pathological situations, including cancer conditions.

While one of the complexes, **2**, according to DPH time-resolved fluorescence intensity and anisotropy decays apparently led to a fluidization of the membrane and increased hydration (as observed for the simple systems) the changes induced by **1** could be better explained by a reorganization of membrane domains. To get more insight into the effect of these complexes in cancer cells, membrane dipole potential was also measured. The dipole potential affects the activity of many membrane proteins and is strongly dependent on the levels and interaction of cholesterol and sphingolipids, which are changed in human tumor cells. The presence of both complexes increased the dipole potential in the **C-model**, while no effect was detected on the **N-model**.

However, the mechanism by which the dipole potential was elevated should be different for each compound, a view that is supported by dipole potential measurements in DOPC bilayers. In conclusion, in the complex membrane systems, the impact of each ruthenium complex was distinct, and maybe more importantly, a clear effect that was not observed in the mixture mimicking normal cells was clearly detected on the mixture mimicking cancer cells.

Finally, the lipid domains reorganization induced by **1** seems to be occurring mostly at the *L*_*o*_ domains level, i.e., the domains that mimic lipid raft domains in mammalian cell membranes, since the effect was much more pronounced when reported by di-4-ANEPPS, a probe that prefers *L*_*o*_ domains through a parameter that is strongly affected by sphingolipid/cholesterol interactions, than that reported by DPH, a probe with no specific preference for *L*_*d*_ or *L*_*o*_ domains.

The decrease in lipid rafts, mainly through decreased cholesterol levels, has already been shown to have drastic effects on cancer cells (Li et al., 2006; Vona et al., 2021). It would not be surprising if an analogous effect was observed when the biophysical properties of the rafts undergo changes, since these have effects that are not only related to cell proliferation and death, but also to the metastatic process (Murai, 2014; Maja et al., 2022). The manner how (and to what extent) lipids and proteins involved in membrane domain organization are affected by active Ru complexes is certainly a subject that motivates further research. This work highlights that the cell membrane biophysical properties may play an important role in the mode of action of these ruthenium complexes.

## Data Availability

The original contributions presented in the study are included in the article/Supplementary Material, further inquiries can be directed to the corresponding authors.

## Abbreviations

CF, 5(6)-carboxyfluorescein; Chol, cholesterol; di-4-ANEPPS, 4-(2-(6-(dibutylamino)-2-naphthalenyl)ethenyl)-1-(3-sulfopropyl)pyridinium hydroxide inner salt; DMPC, 1,2-dimyristoyl-*sn*-glycero-3-phosphocholine; DMPG, 1,2-dimyristoyl-*sn*-glycero-3-phosphoglycerol; DOPC, 1,2-dioleoyl-*sn*-glycero-3-phosphocholine; DPH, 1,6-diphenyl-1,3,5-hexatriene; DPPC, 1,2-dipalmitoyl-*sn*-glycero-3-phosphocholine; LUVs, large unilamellar vesicles; POPC, 1-palmitoyl-2-oleoyl-*sn*-glycero-3-phosphocholine; POPE, 1-palmitoyl-2-oleoyl-*sn*-Glycero-3-phosphoethanolamine; *Salan*, class of tetrahydro-*salen* compounds, i.e., compounds with the *salen* skeleton reduced at the imine function; *Salen*, class of compounds based on the *N*,*N*′-bis(salicylidene)-1,2-ethylenediamine skeleton; SM, sphingomyelin.
