# Supplementary material for: Assessing the role of membrane lipids in the action of ruthenium(III) anticancer compounds
Source: Front Mol Biosci. 2023 Jan 4;9:1059116. doi: 10.3389/fmolb.2022.1059116 (PMC9845782; doi:10.3389/fmolb.2022.1059116)
Supplement: Supplementary file 1 [file DataSheet1.pdf]

Assessing the role of membrane lipids in the action of ruthenium(III) anticancer compounds.

Supplementary Material

Radosław Starosta[1,2], Telma C. Santos[2], Andreia F. Dinis de Sousa[2], Maria Soledade Santos[2], M. Luísa Corvo[3], Ana Isabel Tomaz[2*]and Rodrigo F. M. de Almeida[2*]

[1] Faculty of Chemistry, University of Wroclaw, Joliot-Curie 14, 50-383 Wroclaw, Poland

[2] Centro de Química Estrutural, Institute of Molecular Sciences, Departamento de Química e Bioquímica, Faculdade de Ciências, Universidade de Lisboa, Campo Grande, 1749-016 Lisbon, Portugal

[3] Research Institute for Medicines (iMed.ULisboa), Faculdade de Farmácia, Universidade de Lisboa, Lisbon 1649-003, Portugal

List of figures and tables:

Supplementary Figure S1. Stability of ligand **L1** during 24 h. **A:** c = 100 µM in *buffer 3* (Hepes 10 mM pH 7,4 with 5% DMSO); **B:** c = 40 µM in *buffer 1* (Hepes 10 mM, NaCl 150 mM, pH 7,4 with 5% DMSO). Absorption spectra with zoomed 260-300 nm region and percentage variation of the absorbance at 277 nm. - **page 3**

Supplementary Figure S2. Stability of ligand **L2** during 24 h. **A:** c = 100 µM in *buffer 3* (Hepes 10 mM pH 7,4 with 5% DMSO); **B:** c = 40 µM in *buffer 1* (Hepes 10 mM, NaCl 150 mM, pH 7,4 with 5% DMSO). Absorption spectra with zoomed 260-320 nm region and percentage variation of the absorbance at 290 nm. - **page 4**

Supplementary Figure S3. Stability of complex **1** during 24 h. c(**1**) = 55 µM in *buffer 1* (Hepes 10 mM, NaCl 150 mM, pH 7,4 with 5% DMSO). Normalized absorption spectra with zoomed 750-850 nm region and percentage variation of the absorbance at 850 nm. - **page 5**

Supplementary Figure S4. Stability of complex **1** during 24 h in (**A**) *buffer 2* (c(**1**) = 55 µM; Hepes 10 mM pH 7,4 with 2% DMSO) and (**B**) *buffer 2* (c(**1**) = 25 µM) in the presence of DOPC LUVs. Absorption spectra with zoomed 320-380 and 520-720 nm regions or 300-400 and 520-720 nm regions; and % variation of the absorbance at 350 and 600 nm or at 350 and 622 nm. - **page 6**

Supplementary Figure S5. Stability of complex **2** over 24 h in the presence of DOPC LUVs in (**A**) *buffer 2* (c(**1**) = 61 µM; Hepes 10 mM pH 7,4 with 2% DMSO), and in (**B**) *buffer 2* (c(**1**) = 27 µM).

Absorption spectra with zoomed 280-350 nm and 550-750 nm regions or 280-450 and 550-720 nm regions; and % variation of the absorbance at 314 and 650 nm. - **page 7**

Supplementary Figure S6. Emission spectra of di-4-ANEPPS in DOPC LUVs in the presence of increasing concentration of 1 (A) and of 2 (B) as indicated by the arrow, with the corresponding Stern-Volmer curves for 1 (C) and 2 (D) fitted to the polynomial curves as indicated. [DOPC] = 1.5 mM) - **page 8**

Supplementary Figure S7. Mole fraction (%) of compound effectively present in the DOPC bilayer lipid phase as a function of the lipid concentration calculated using the equation in *inset* (Eq. 7 in the main text). - **page 9**

Supplementary Figure S8. Excitation (---) and emission (–) spectra of DPH labelling **C-model** (POPC:POPE:SM:Chol 36.2:23.6:6.8:33.4) in the absence (control, blue) and in the presence of complexes **1** (on the left) and **2** (on the right). **A**, **B** and **C** correspond to fluorescence intensity, normalized fluorescence intensity, and the background percentage, respectively. - **page 10**

Supplementary Figure S9. Fluorescence decay curves of DPH labelling DOPC, DPPC, **N-model** and **C-model** *LUVs* in the absence (blue, control) and presence of the complexes **1** (green) and **2** (yellow). - **page 11**

Supplementary Table S1. Fluorescence spectroscopic properties of DPH in DOPC, DPPC, **N-model** and **C-model** LUVs in the absence (control; black) and presence of the complexes **1** (blue) and **2** (red). - **pages 12-13**

Supplementary Figure S10. Excitation and emission spectra before (left) and after (right) peak normalization, and fluorescence decay curves of di-4-ANEPPS labelling **N-model** or **C-model** LUVs in the absence (blue) and presence of the complexes **1** (green) and **2** (yellow). - **page 14**

Supplementary Figure S11. Fluorescence properties of di-4-ANEPPS labelling DOPC LUVs. **A:** normalized excitation and emission spectra of di-4-ANEPPS in the absence (black) and presence (orange) of **1** (**A:1**) and **2** (**A:2**); **B:** intensity-weighted mean fluorescence lifetime <τ>, and **C:** steady-state fluorescence anisotropy <r> of the probe in the absence (control) and presence of **1** and **2** (*buffer 3*: Hepes 10 mM pH 7,4 with 5% DMSO; c(**1**) = c(**2**) = 20 µM). - **page 15**

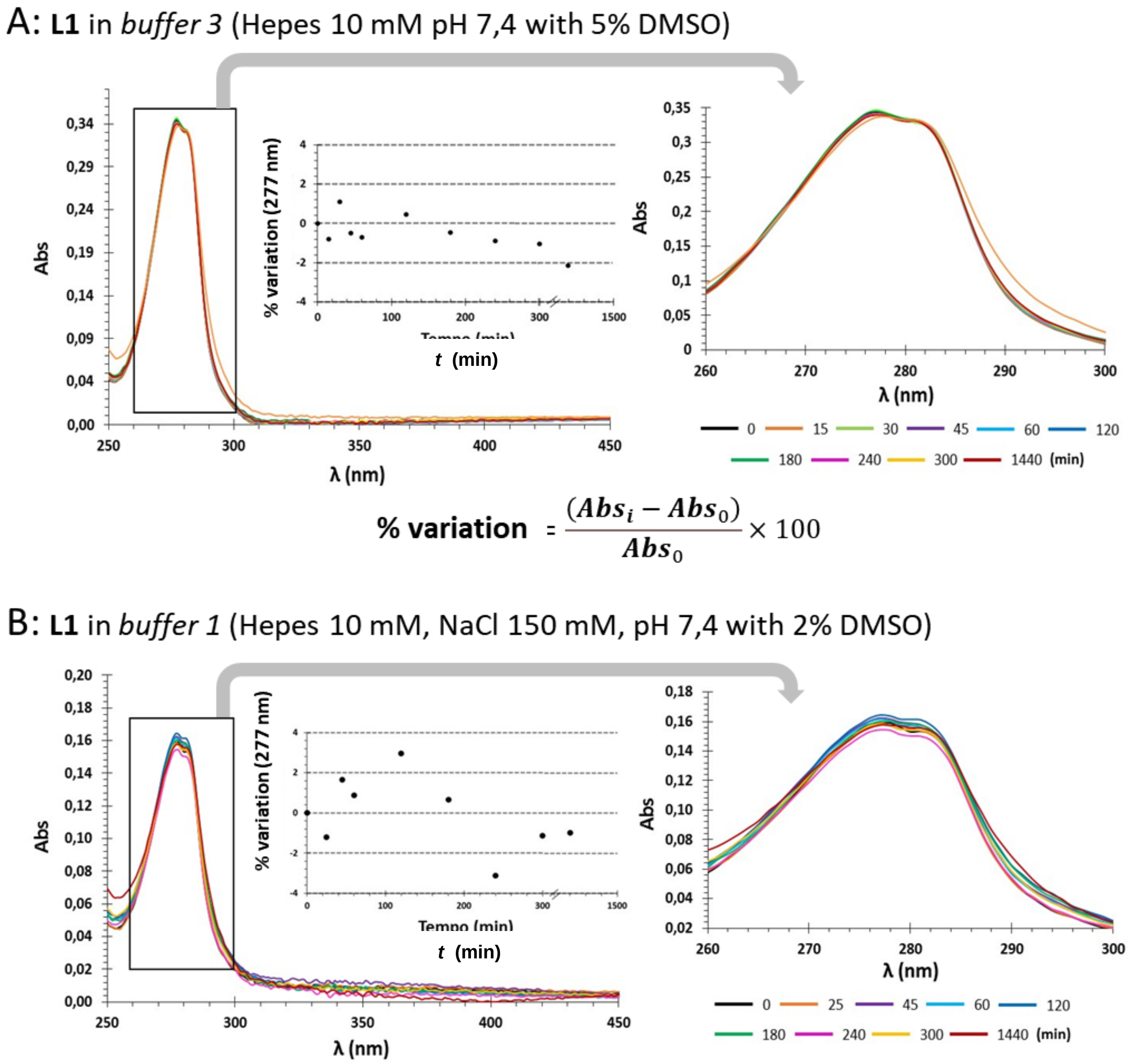

Supplementary Figure S1. Stability of ligand **L1** during 24 h. **A:** c = 100 µM in *buffer 3* (Hepes 10 mM pH 7,4 with 5% DMSO); **B:** c = 40 µM in *buffer 1* (Hepes 10 mM, NaCl 150 mM, pH 7,4 with 5% DMSO). Absorption spectra with zoomed 260-300 nm region and percentage variation of the absorbance at 277 nm.

A: **L2** in *buffer 3* (Hepes 10 mM pH 7,4 with 5% DMSO)

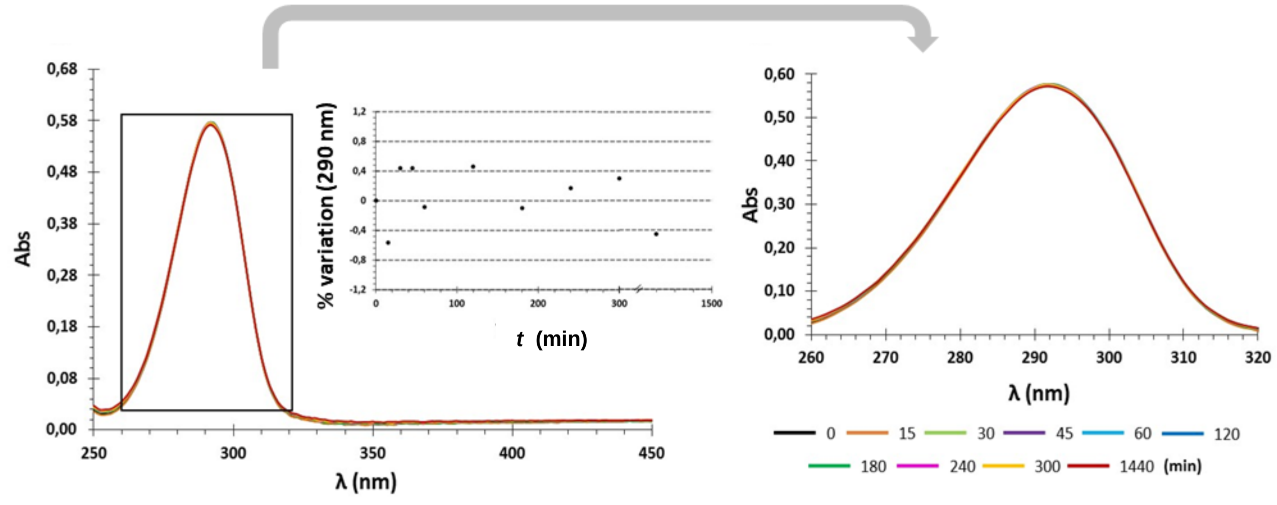

$$\% \text{ variation} = \frac{(Abs_i - Abs_0)}{Abs_0} \times 100$$

B: **L2** in *buffer 1* (Hepes 10 mM, NaCl 150 mM, pH 7,4 with 2% DMSO)

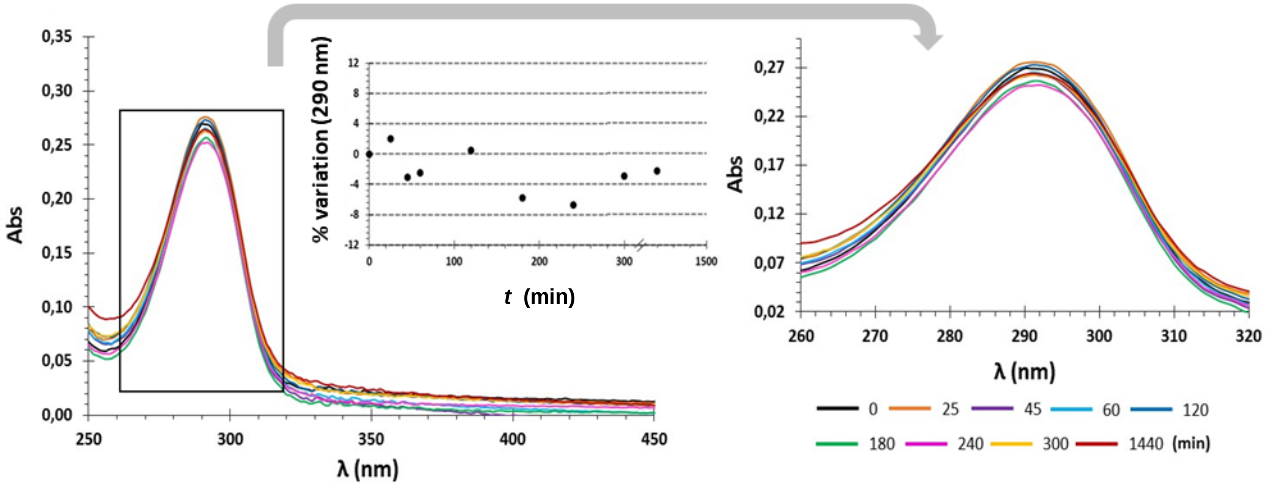

Supplementary Figure S2. Stability of ligand **L2** during 24 h. **A:** c = 100 μM in *buffer 3* (Hepes 10 mM pH 7,4 with 5% DMSO); **B:** c = 40 μM in *buffer 1* (Hepes 10 mM, NaCl 150 mM, pH 7,4 with 5% DMSO). Absorption spectra with zoomed 260-320 nm region and percentage variation of the absorbance at 290 nm.

1 in *buffer 1* (Hepes 10 mM, NaCl 150 mM, pH 7,4 with 2% DMSO)

$$\textbf{\% variation} = \frac{(Abs_i - Abs_0)}{Abs_0} \times 100$$

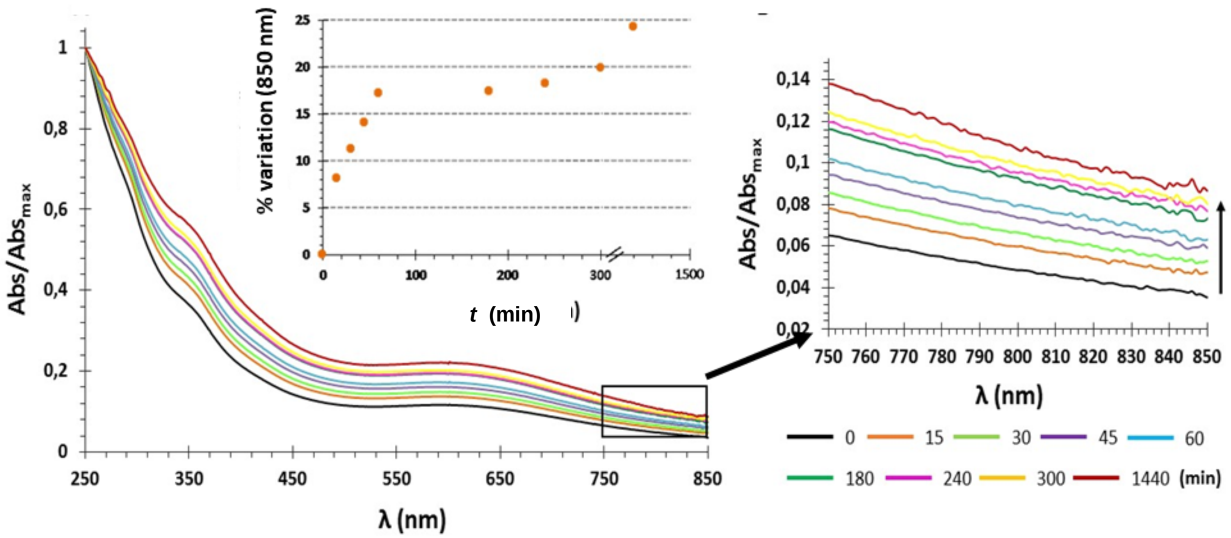

Supplementary Figure S3. Stability of complex **1** during 24 h. c(**1**) = 55 µM in *buffer 1* (Hepes 10 mM, NaCl 150 mM, pH 7,4 with 5% DMSO). Normalized absorption spectra with zoomed 750-850 nm region and percentage variation of the absorbance at 850 nm.

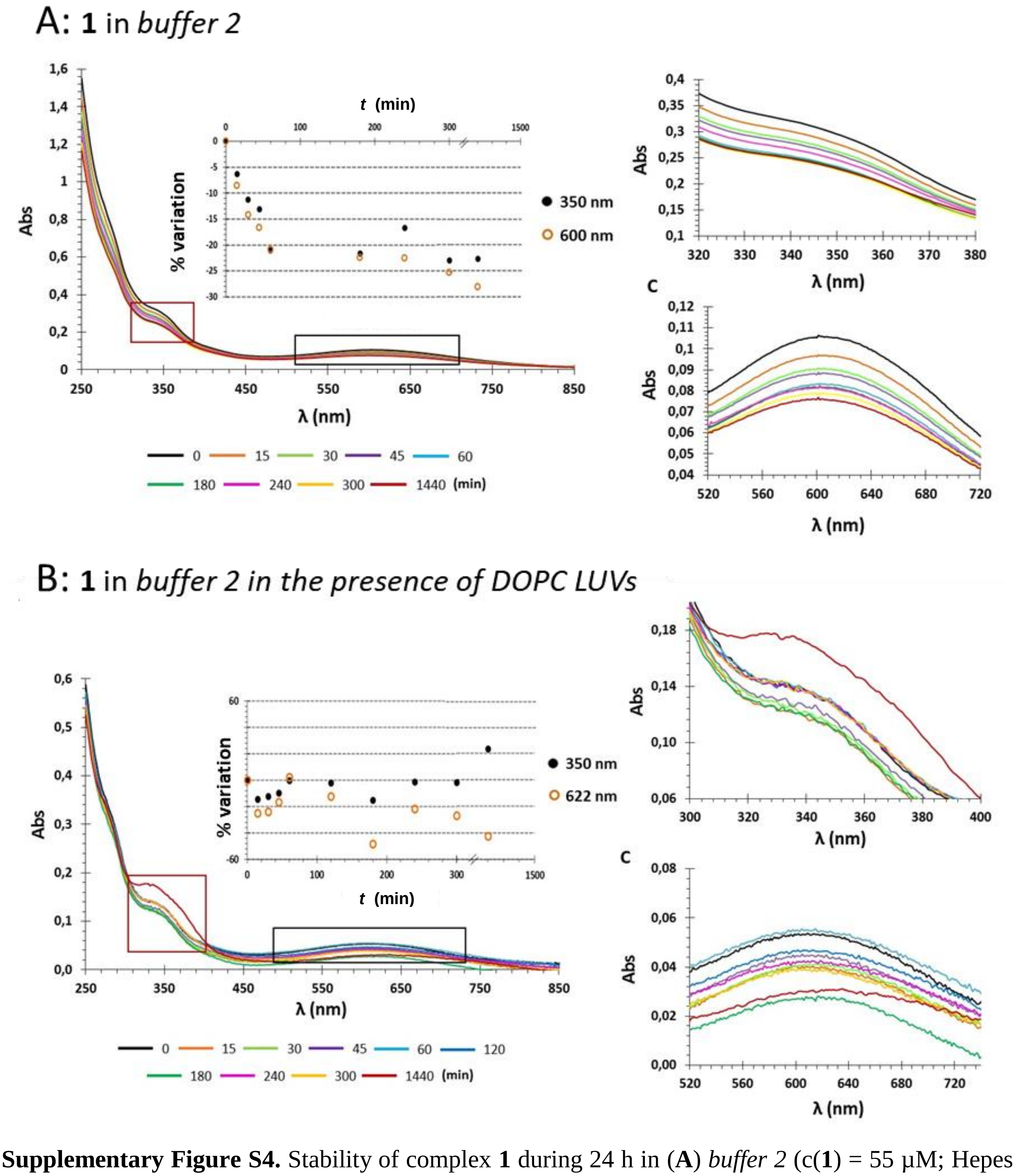

Supplementary Figure S4. Stability of complex **1** during 24 h in (**A**) *buffer 2* (c(**1**) = 55 µM; Hepes 10 mM pH 7,4 with 2% DMSO) and (**B**) *buffer 2* (c(**1**) = 25 µM) in the presence of DOPC LUVs. Absorption spectra with zoomed 320-380 and 520-720 nm regions or 300-400 and 520-720 nm regions; and % variation of the absorbance at 350 and 600 nm or at 350 and 622 nm.

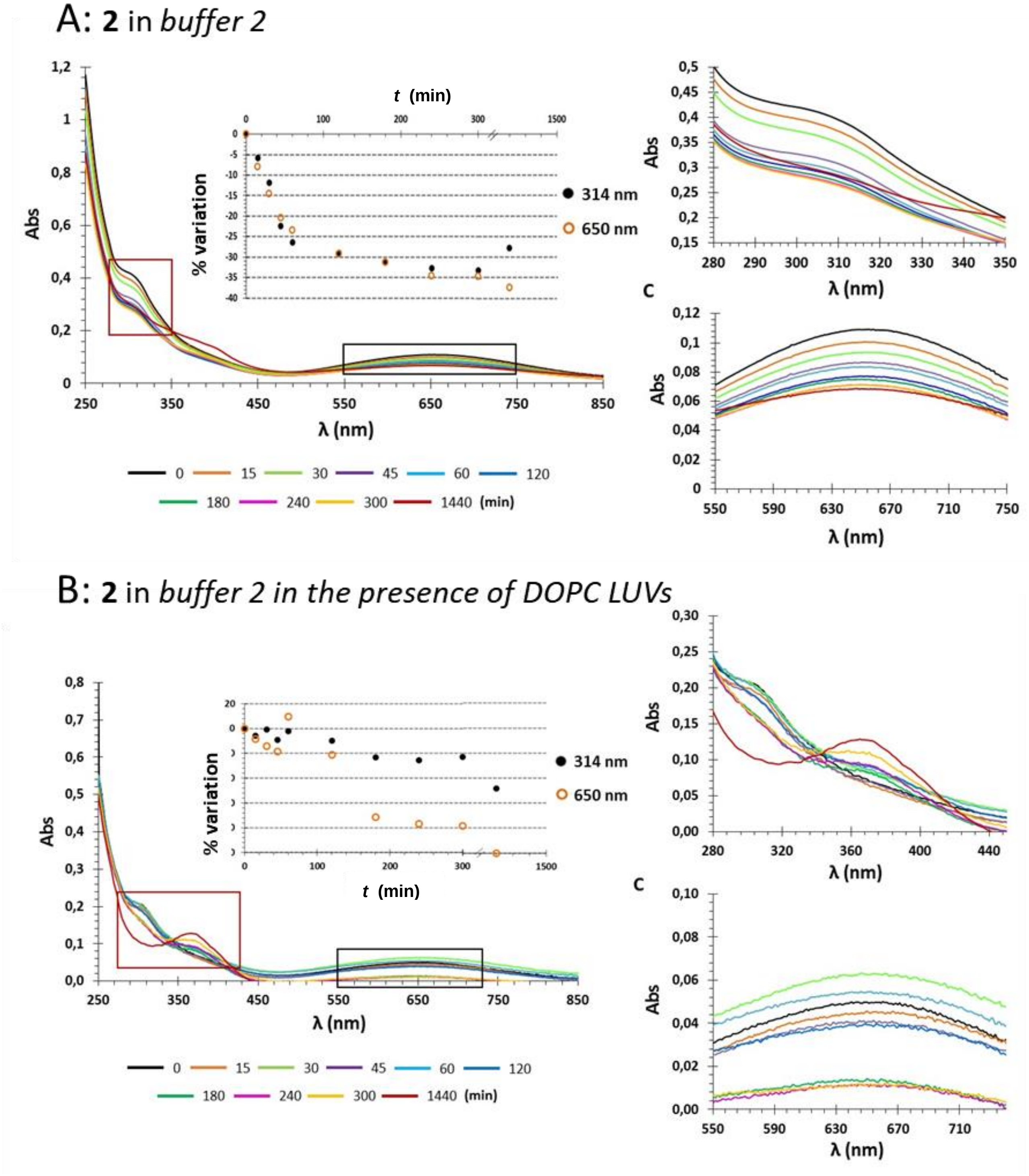

Supplementary Figure S5. Stability of complex **2** over 24 h in the presence of DOPC LUVs in (**A**) *buffer 2* (c(**1**) = 61 µM; Hepes 10 mM pH 7,4 with 2% DMSO), and in (**B**) *buffer 2* (c(**1**) = 27 µM). Absorption spectra with zoomed 280-350 and 550-750 nm regions or 280-450 and 550-720 nm regions; and % variation of the absorbance at 314 and 650 nm.

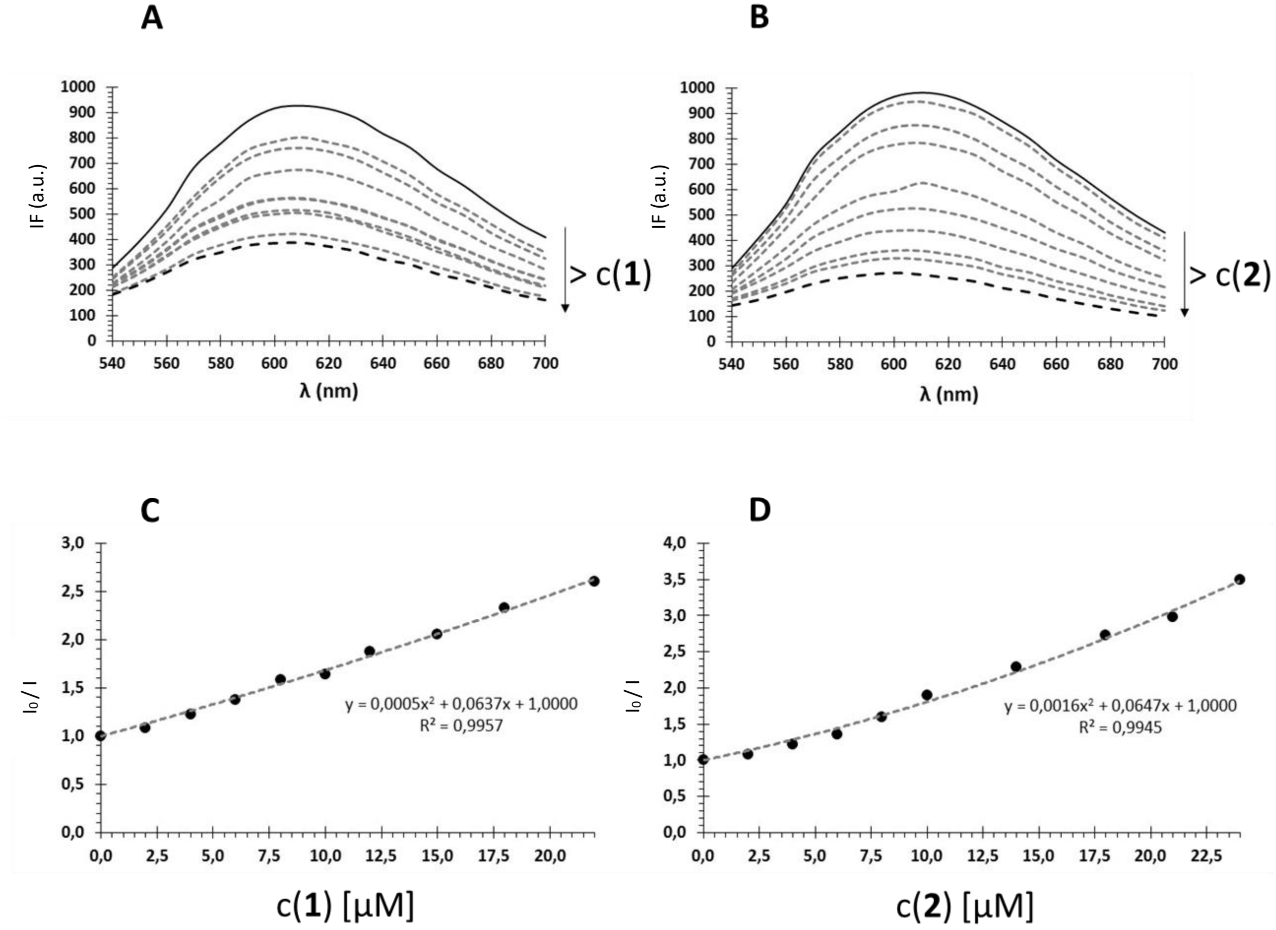

Supplementary Figure S6. Emission spectra of di-4-ANEPPS in DOPC LUVs in the presence of increasing concentration of **1** (A) and of **2** (B) as indicated by the arrow, with the corresponding Stern-Volmer curves for **1** (C) and **2** (D) fitted to the polynomial curves as indicated. [DOPC] = 1.5 mM)

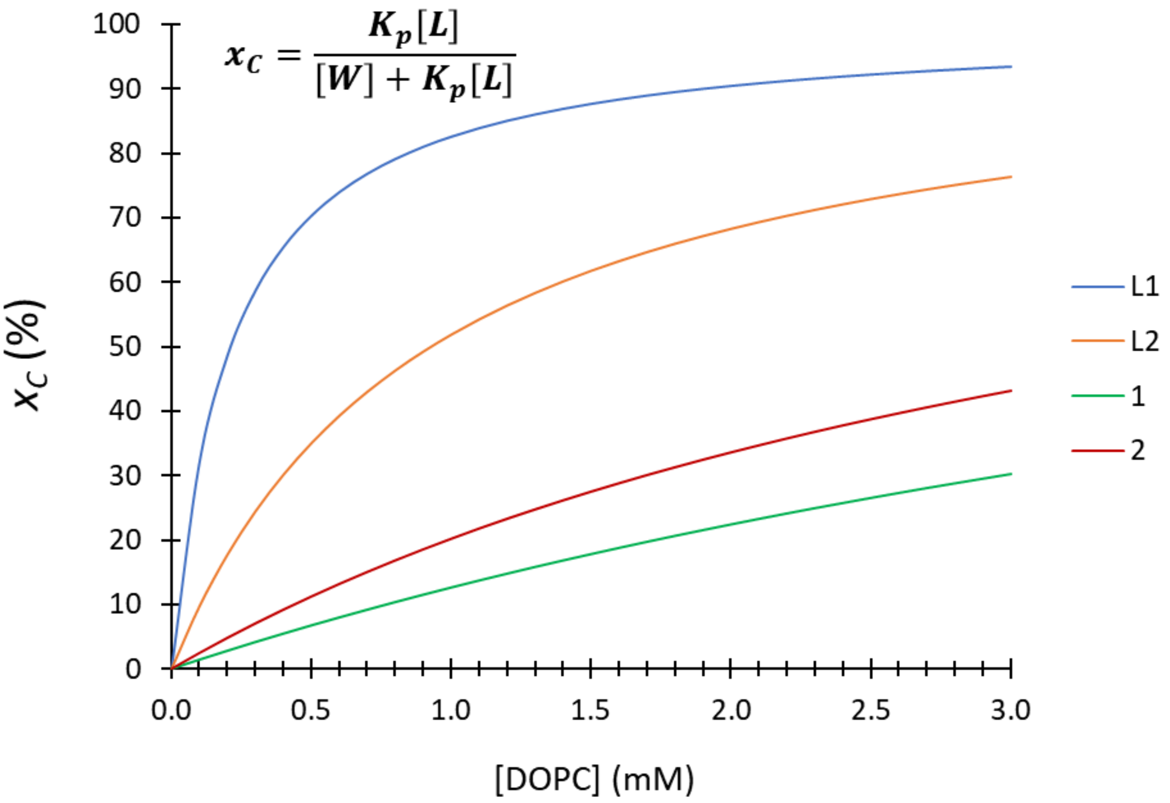

Supplementary Figure S7. Mole fraction (%) of compound effectively present in the DOPC bilayer lipid phase as a function of the lipid concentration calculated using the equation in *inset* (Eq. 7 in the main text).

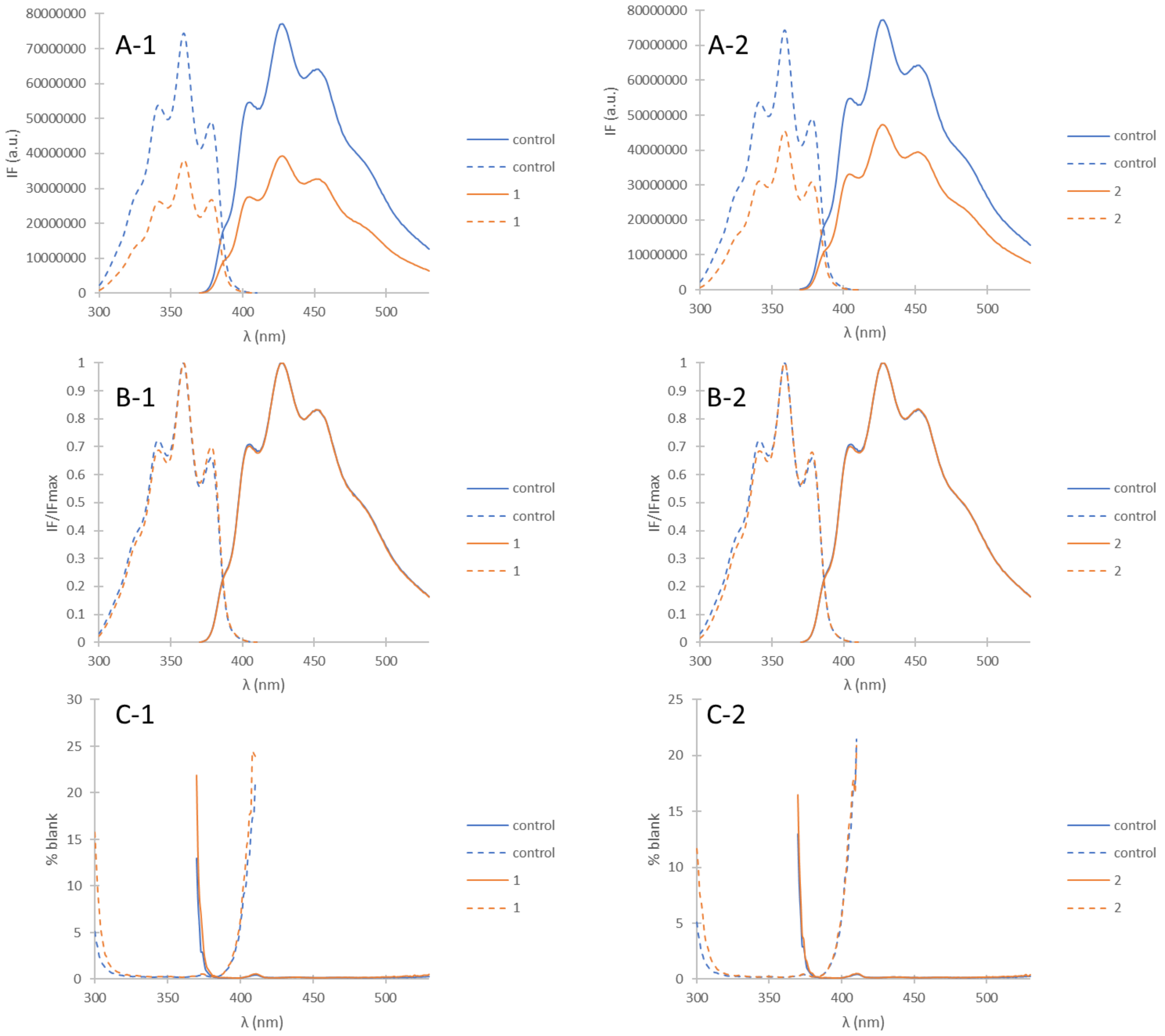

Supplementary Figure S8. Excitation (---) and emission (–) spectra of DPH labelling **C-model** (POPC:POPE:SM:Chol 36.2:23.6:6.8:33.4) in the absence (control, blue) and in the presence of complexes **1** (on the left) and **2** (on the right). A, B and C correspond to fluorescence intensity, normalized fluorescence intensity, and the background percentage, respectively.

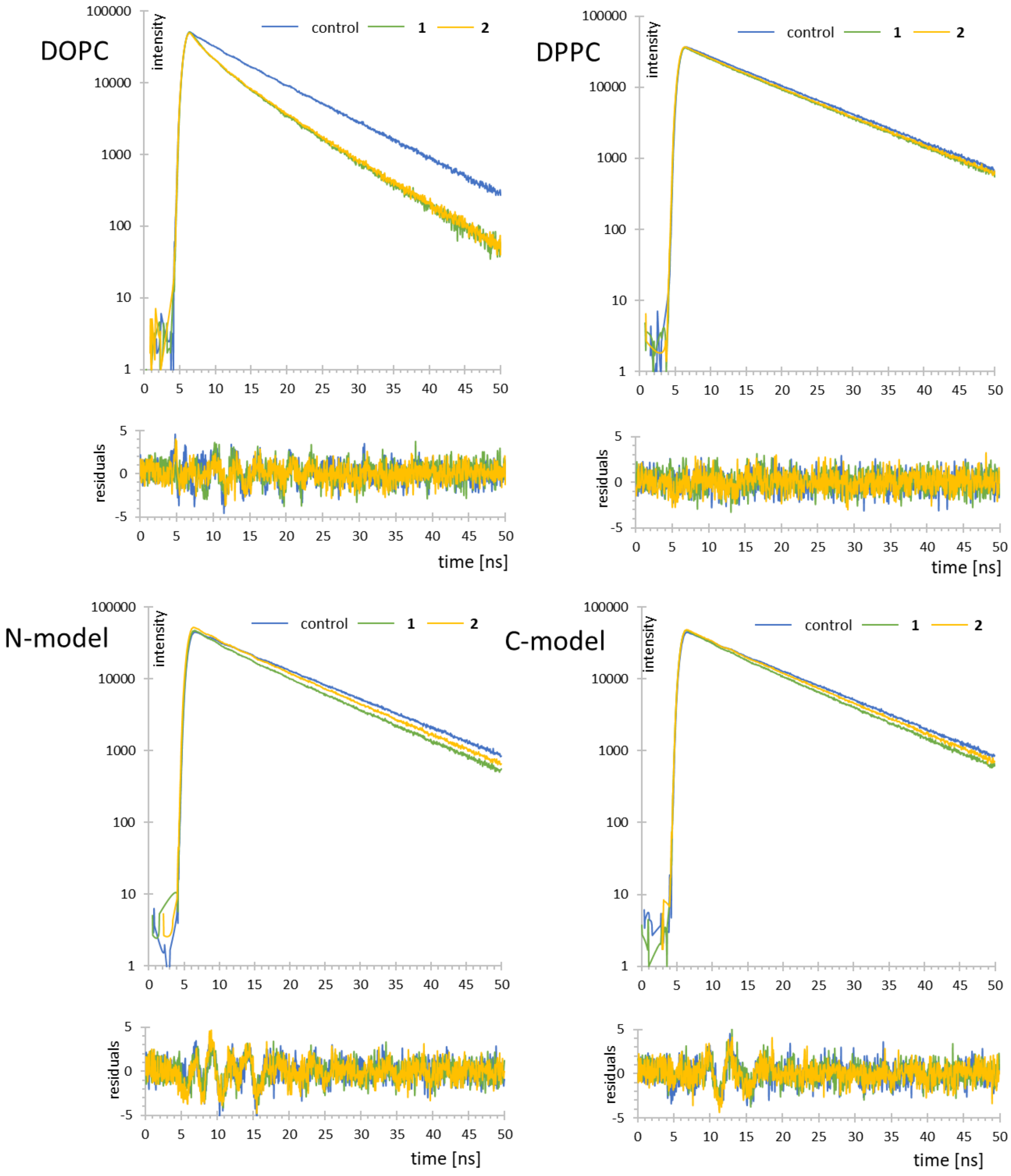

Supplementary Figure S9. Fluorescence decay curves of DPH labelling DOPC, DPPC, **N-model** and **C-model** LUVs in the absence (blue, control) and presence of the complexes **1** (green) and **2** (yellow).

Supplementary Table S1. Fluorescence spectroscopic properties of DPH in DOPC, DPPC, **N-model** and **C-model** LUVs in the absence (control; black) and presence of the complexes **1** (blue) and **2** (red). (see Materials and Methods in the main text for more information)

	DOPC		DPPC		N-model		C-model		
	**av.value**	st.dev.	**av.value**	st.dev.	**av.value**	st.dev.	**av.value**	st.dev.	
**τ_1 (ns)**	**0.87**	0.07							**ctrl**
	**1.17**	0.07	**1.25**	0.13	**3.42**	0.41	**3.36**	0.22	**1**
	**0.70**	0.02	**0.52**	0.07	**0.22**	0.04	**0.23**	0.05	**2**
**τ_2 (ns)**	**3.38**	0.14	**4.90**	0.12	**7.02**	0.89	**5.69**	0.37	**ctrl**
	**3.50**	0.15	**4.54**	0.11	**8.88**	0.27	**9.15**	0.43	**1**
	**3.08**	0.07	**4.32**	0.07	**4.46**	0.07	**4.01**	0.41	**2**
**τ_3 (ns)**	**8.55**	0.07	**11.08**	0.11	**11.30**	0.28	**11.03**	0.06	**ctrl**
	**7.19**	0.08	**10.92**	0.08	**11.29**	0.19	**11.84**	0.59	**1**
	**7.26**	0.05	**10.88**	0.08	**10.55**	0.10	**10.57**	0.11	**2**
**α_1**	**0.146**	0.047							**ctrl**
	**0.284**	0.032	**0.029**	0.005	**0.186**	0.012	**0.144**	0.015	**1**
	**0.298**	0.013	**0.060**	0.033	**0.198**	0.038	**0.094**	0.048	**2**
**α_2**	**0.115**	0.008	**0.083**	0.006	**0.074**	0.020	**0.076**	0.012	**ctrl**
	**0.346**	0.010	**0.122**	0.008	**0.406**	0.033	**0.394**	0.123	**1**
	**0.340**	0.014	**0.118**	0.011	**0.152**	0.012	**0.146**	0.022	**2**
**α_3**	**0.739**	0.015	**0.917**	0.006	**0.926**	0.106	**0.924**	0.022	**ctrl**
	**0.370**	0.042	**0.849**	0.029	**0.408**	0.034	**0.462**	0.101	**1**
	**0.363**	0.014	**0.822**	0.024	**0.649**	0.066	**0.759**	0.030	**2**
**f_1**	**0.019**	0.004							**ctrl**
	**0.079**	0.020	**0.004**	0.001	**0.072**	0.001	**0.051**	0.003	**1**
	**0.053**	0.005	**0.003**	0.001	**0.006**	0.002	**0.003**	0.002	**2**
**f_2**	**0.057**	0.009	**0.039**	0.003	**0.047**	0.005	**0.040**	0.014	**ctrl**
	**0.288**	0.030	**0.056**	0.003	**0.407**	0.036	**0.377**	0.134	**1**
	**0.269**	0.015	**0.054**	0.002	**0.090**	0.004	**0.068**	0.003	**2**
**f_3**	**0.925**	0.009	**0.961**	0.003	**0.953**	0.036	**0.960**	0.012	**ctrl**
	**0.633**	0.050	**0.940**	0.004	**0.521**	0.037	**0.572**	0.151	**1**
	**0.677**	0.019	**0.943**	0.000	**0.904**	0.002	**0.930**	0.002	**2**
**$\langle\tau\rangle_a$**	**6.84**	0.10	**10.57**	0.08	**10.98**	0.05	**10.62**	0.21	**ctrl**
**(ns)**	**4.20**	0.24	**9.86**	0.28	**8.85**	0.36	**9.56**	0.14	**1**
	**3.88**	0.03	**9.48**	0.25	**7.57**	0.36	**8.63**	0.25	**2**
**$\langle\tau\rangle$**	**8.12**	0.03	**10.84**	0.05	**11.10**	0.06	**10.81**	0.02	**ctrl**
**(ns)**	**5.65**	0.21	**10.52**	0.03	**9.74**	0.15	**10.40**	0.08	**1**
	**5.78**	0.07	**10.49**	0.04	**9.94**	0.10	**10.10**	0.12	**2**

Supplementary Table S1 (continued). Fluorescence spectroscopic properties of DPH in DOPC, DPPC, **N-model** and **C-model** LUVs in the absence (control; black) and presence of the complexes **1** (blue) and **2** (red) (see Materials and Methods in the main text for more information).

	DOPC		DPPC		N-model		C-model		
	**av.value**	dev.	**av.value**	dev.	**av.value**	dev.	**av.value**	dev.	
β_1	**0.090**	0.006	**0.017**	0.002	**0.053**	0.001	**0.063**	0.008	**ctrl**
	**0.099**	0.017	**0.014**	0.005	**0.044**	0.004	**0.059**	0.009	**1**
	**0.100**	0.008	**0.014**	0.002	**0.052**	0.002	**0.064**	0.010	**2**
β_2	**0.234**	0.007	**0.010**	0.003	**0.051**	0.002	**0.045**	0.008	**ctrl**
	**0.218**	0.015	**0.008**	0.001	**0.036**	0.003	**0.029**	0.006	**1**
	**0.239**	0.008	**0.007**	0.002	**0.046**	0.004	**0.028**	0.006	**2**
r_∞			**0.303**	0.002	**0.214**	0.003	**0.214**	0.007	**ctrl**
	**0.006**	0.001	**0.306**	0.003	**0.210**	0.002	**0.205**	0.002	**1**
	**0.005**	0.000	**0.312**	0.002	**0.206**	0.004	**0.204**	0.003	**2**
<r>	**0.086**	0.002	**0.309**	0.002	**0.232**	0.001	**0.222**	0.002	**ctrl**
	**0.118**	0.001	**0.310**	0.003	**0.224**	0.003	**0.221**	0.003	**1**
	**0.113**	0.003	**0.315**	0.002	**0.223**	0.002	**0.219**	0.002	**2**
Φ_1 (ns)	**0.82**	0.05	**0.49**	0.27	**0.44**	0.03	**0.57**	0.07	**ctrl**
	**0.78**	0.21	**0.61**	0.18	**0.69**	0.18	**0.73**	0.03	**1**
	**0.44**	0.10	**0.49**	0.08	**0.56**	0.08	**0.94**	0.07	**2**
Φ_2 (ns)	**3.74**	0.08	**12.28**	0.98	**4.30**	0.08	**4.16**	0.56	**ctrl**
	**4.03**	0.22	**5.67**	1.12	**4.33**	0.71	**4.79**	0.61	**1**
	**3.43**	0.15	**8.37**	0.36	**3.45**	0.35	**4.47**	0.65	**2**

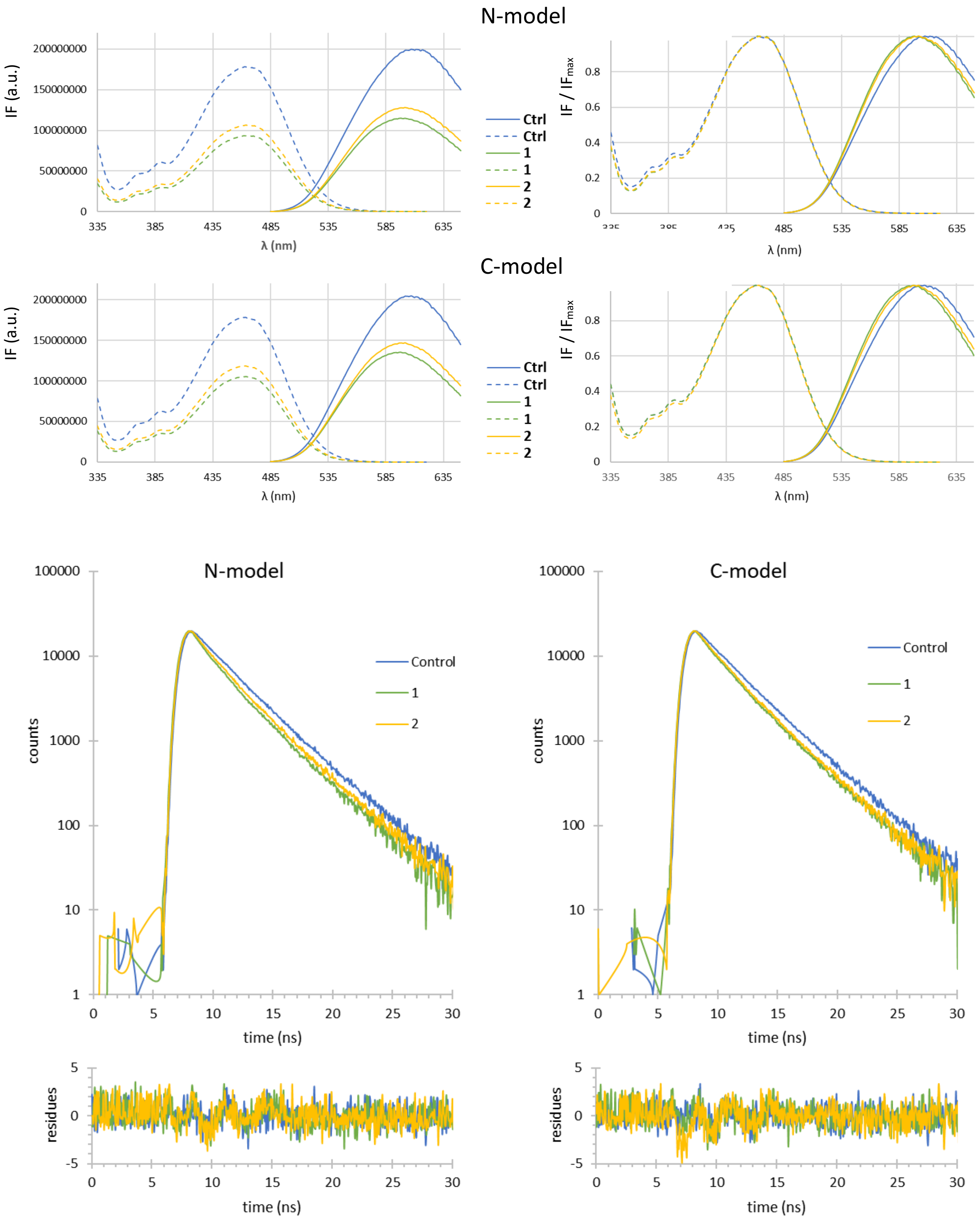

Supplementary Figure S10. Excitation and emission spectra before (left) and after (right) peak normalization, and fluorescence decay curves of di-4-ANEPPS labelling **N-model** or **C-model** LUVs in the absence (blue) and presence of the complexes **1** (green) and **2** (yellow).

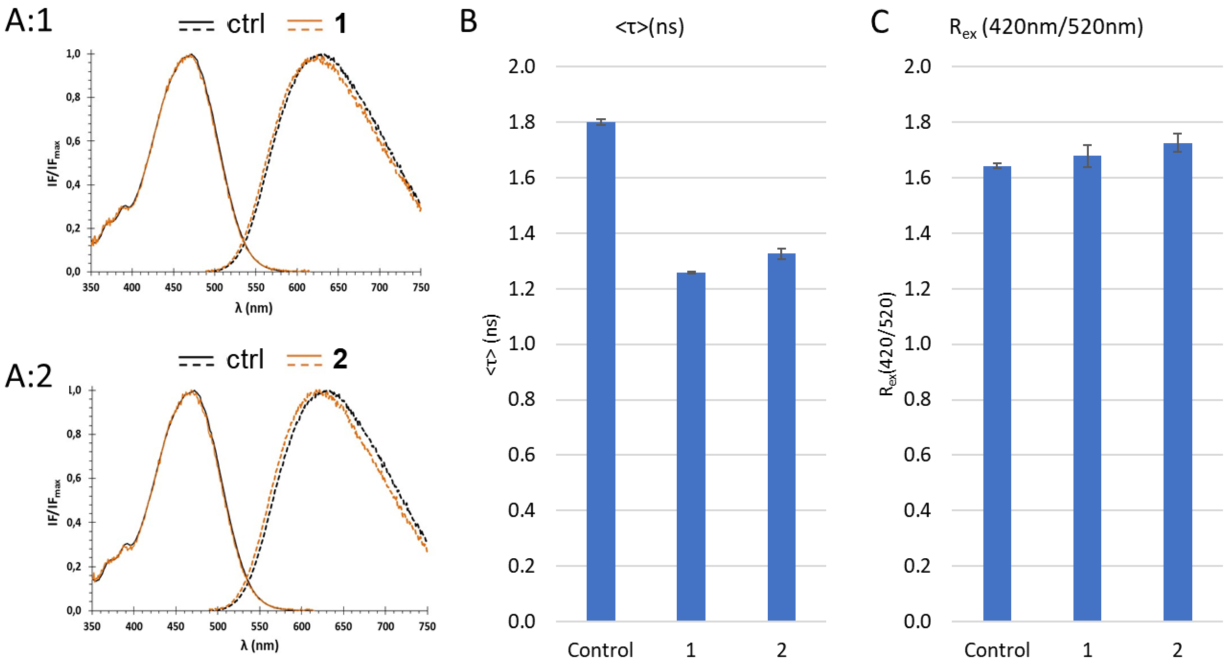

Supplementary Figure S11. Fluorescence properties of di-4-ANEPPS labelling DOPC LUVs. **A:** normalized excitation and emission spectra of di-4-ANEPPS in the absence (black) and presence (orange) of **1** (**A:1**) and **2** (**A:2**); **B:** intensity-weighted mean fluorescence lifetime <τ>, and **C:** steady-state fluorescence anisotropy <r> of the probe in the absence (control) and presence of **1** and **2** (*buffer 3*: Hepes 10 mM pH 7,4 with 5% DMSO; c(**1**) = c(**2**) = 20 µM).
